# Multivalent insulin receptor activation using insulin–DNA origami nanostructures

**DOI:** 10.1038/s41565-023-01507-y

**Published:** 2023-10-09

**Authors:** Joel Spratt, José M. Dias, Christina Kolonelou, Georges Kiriako, Enya Engström, Ekaterina Petrova, Christos Karampelias, Igor Cervenka, Natali Papanicolaou, Antonio Lentini, Björn Reinius, Olov Andersson, Elena Ambrosetti, Jorge L. Ruas, Ana I. Teixeira

**Affiliations:** 1 Department of Medical Biochemistry and Biophysics, Karolinska Institutet, Stockholm, Sweden; 2 Department of Cell and Molecular Biology, Karolinska Institutet, Stockholm, Sweden; 3 Department of Physiology and Pharmacology, Karolinska Institutet, Stockholm, Sweden; 4 Center for Life Nano- and Neuro-Science, Istituto Italiano di Tecnologia, Rome, Italy

**Keywords:** Nanobiotechnology, DNA nanostructures

## Abstract

Insulin binds the insulin receptor (IR) and regulates anabolic processes in target tissues. Impaired IR signalling is associated with multiple diseases, including diabetes, cancer and neurodegenerative disorders. IRs have been reported to form nanoclusters at the cell membrane in several cell types, even in the absence of insulin binding. Here we exploit the nanoscale spatial organization of the IR to achieve controlled multivalent receptor activation. To control insulin nanoscale spatial organization and valency, we developed rod-like insulin–DNA origami nanostructures carrying different numbers of insulin molecules with defined spacings. Increasing the insulin valency per nanostructure markedly extended the residence time of insulin–DNA origami nanostructures at the receptors. Both insulin valency and spacing affected the levels of IR activation in adipocytes. Moreover, the multivalent insulin design associated with the highest levels of IR activation also induced insulin-mediated transcriptional responses more effectively than the corresponding monovalent insulin nanostructures. In an in vivo zebrafish model of diabetes, treatment with multivalent—but not monovalent—insulin nanostructures elicited a reduction in glucose levels. Our results show that the control of insulin multivalency and spatial organization with nanoscale precision modulates the IR responses, independent of the insulin concentration. Therefore, we propose insulin nanoscale organization as a design parameter in developing new insulin therapies.

## Abstract (web summary)

DNA-origami-based insulin assembly into well-defined nanoclusters reveals that insulin valency and spatial organization modulate insulin receptor activation and downstream responses independent of ligand concentration.

## Main

The peptide hormone insulin has been the subject of intense research since its discovery by Banting and Best over 100 years ago, with profound medical implications^1^. The fundamental mechanisms of insulin action are now well established. Binding of insulin to the insulin receptor (IR) induces conformational changes that lead to receptor autophosphorylation, initiating a signalling cascade that culminates in glucose uptake into cells, lipogenesis, glycogenesis and other anabolic, biosynthetic processes^1,2^. The structural details of insulin binding and the associated IR conformational changes have been elucidated in recent years^3–5^. Interestingly, IRs have been reported to form nanoclusters at the cell membrane since the 1970s, for example, in adipocytes^6,7^, β-cells^8^ and hepatocytes^9^. However, the conceptual focus of research on IR has primarily been on single ligand–receptor interactions, with little consideration for the broader nanoscale organization of receptors at the cell membrane.

Targeting receptor nanodomains with well-defined ligand nanoclusters, instead of individual ligands, offers entirely new possibilities to control receptor-mediated signalling responses. DNA origami nanostructures have been used to control the nanoscale spatial distribution of ligands and regulate receptor-mediated signalling in, for example, EPH^10,11^, PD-1 (ref. ^12^), MET^13^, TLR9 (ref. ^14^), FcγR^15^, B-cell^16^ and T-cell^17,18^ receptors. Importantly, DNA origami nanostructures enable the straightforward control of ligand valency, thereby tailoring the generation of avidity effects between nanoclusters of ligands and nanodomains of receptors. Here, using adipocytes as a model system, we characterized the nanoscale spatial organization of IRs at the cell membrane. Further, we used this knowledge to guide the design of well-defined nanoclusters of insulin to explore how insulin valency and spatial organization affect IR signalling both in vitro and in vivo.

## Results

### IRs are organized into nanoclusters

**Fig. 1:**
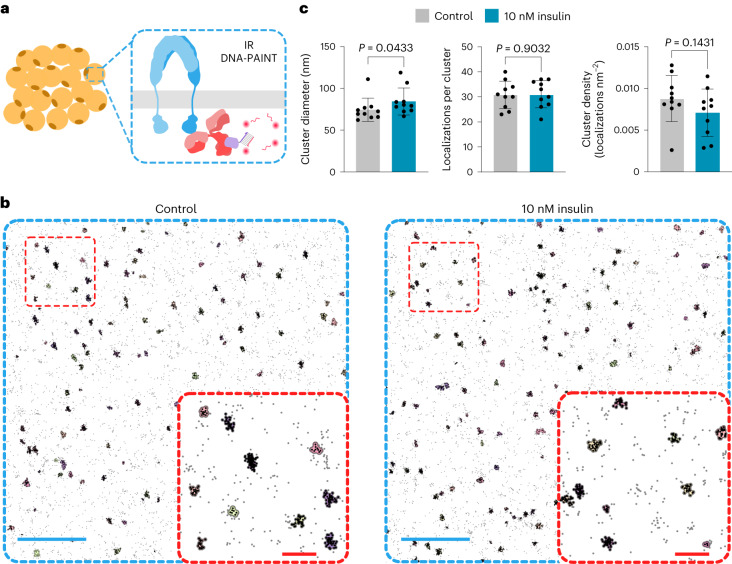
IRs are organized in nanoclusters at the cell membrane. **a**, Schematic of DNA-PAINT used to image IRs (blue) using antibodies (red) targeting the intracellular kinase domain in adipocyte cultures. **b**, Cluster outlines derived from DNA-PAINT of IRs at the cell membrane of adipocytes treated with PBS (control) or with 10 nM of insulin for 10 min. The insets show the magnified regions highlighting the identified clusters. Scale bars, 1 µm (blue); 200 nm (red, inset). **c**, Characterization of IR clusters in control and insulin-treated adipocytes. Data are presented as mean ± standard deviation (s.d.), *n* = 10 cells per condition. The *P* values are determined by a two-tailed Mann–Whitney test. Source data

**Extended Data Fig. 1:**
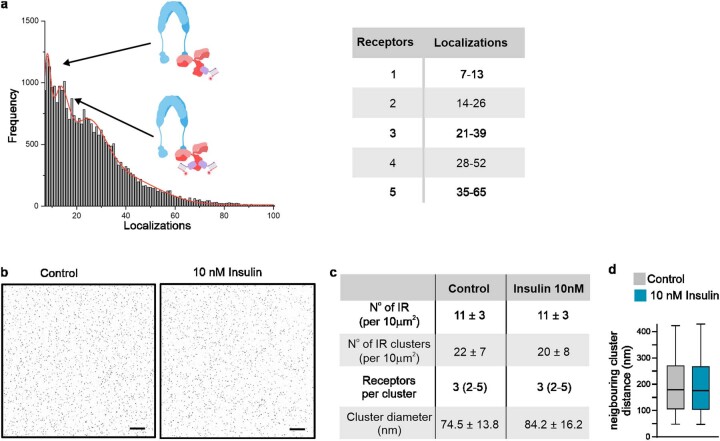
Characterization of insulin receptors at the adipocyte cell membrane. **a**, Histogram of the number of localizations per cluster. Red line represents the Gaussian fit showing multiple peaks representing one, two, three, etc., docking sites. Peaks at 7 ± 0.14 and 13 ± 0.61 both correspond to signal from a single receptor since each primary antibody can be bound by one or two nanobodies conjugated to one DNA-PAINT docking site. The table shows the range of localizations that define the number of receptors. **b**, Insulin receptor cluster outlines in adipocytes treated with 10 nM insulin for 10 mins and corresponding control. Image representative of 3 independent experiments. Scale bar, 2 µm. **c**, Table showing the number of IR and IR clusters per 10 µm^2^ region (mean ± SD), number of receptors per cluster (median and interquartile range), and cluster diameter (mean ± SD) plotted in Fig. 1c, in control and insulin-treated adipocyte cells. **d**, Characterization of the distance between the edges of neighboring IR clusters in Control (median=178.9 nm, 95% CI = 176.1-181.4 nm) and insulin treated (median=175.7 nm, 95% CI = 173.2-178.3 nm) adipocytes. Boxplot data: center line defines median, whiskers indicate 5-95 percentile. n = 16897 (control) and n = 16374 (10 nM Insulin) pairs of clusters examined over 10 cells for each condition. Data points outside whisker limits are omitted from plot. Source data

Among the insulin target cell types, adipocyte cultures offer a biologically relevant, robust and homogeneous system to investigate insulin signalling. To analyse the nanoscale organization of IRs at the cell membrane of cultured adipocytes^19^, we used DNA point accumulation in nanoscale topography (DNA-PAINT)^20^ (Fig. 1a). We found that IRs form nanoclusters with an average diameter of 74 nm (Fig. 1b,c). We observed around 31 localizations per cluster (Fig. 1c), which we estimated to correspond to three to four IRs per cluster, using Bayesian-based cluster analyses^21^ (Extended Data Fig. 1a–c). Treatment with 10 nM insulin for 10 min resulted in a slight increase in the equivalent diameter from 74 to 84 nm (Fig. 1b,c and Extended Data Fig. 1b,c). Although we observed a decrease in the cluster density on insulin treatment, this change was not statistically significant (Fig. 1c). Additionally, IR clusters showed similar distributions at the cell membrane in control and insulin-treated adipocytes, with median distances between the edges of neighbouring clusters of 179 and 176 nm, respectively (Extended Data Fig. 1d). Together, our data confirm that IRs are present in clusters at the cell membrane and this organization is largely maintained after insulin treatment.

### Insulin NanoRods with defined insulin spacings and valencies

**Fig. 2:**
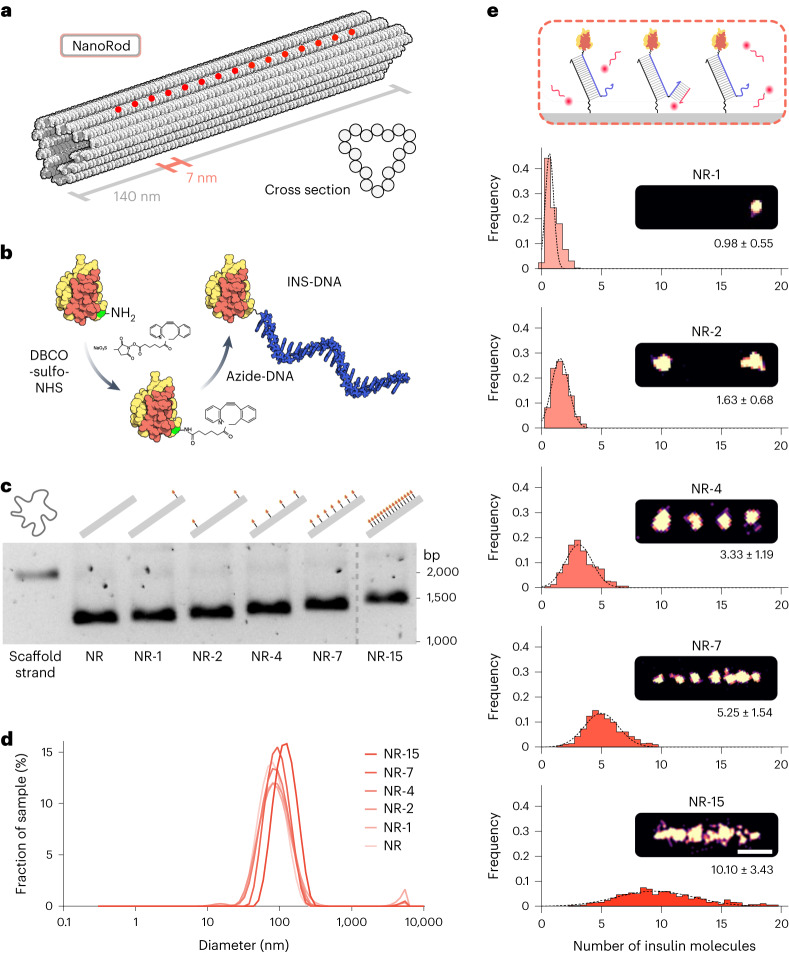
Insulin NanoRods with programmable insulin configurations. **a**, Representation of the DNA origami NanoRod. The red circles correspond to the positions of protruding DNA strands at programmable positions along one of its faces used for hybridization to INS-DNA. **b**, Overview of the method used to conjugate a single azide-modified ssDNA oligonucleotide to the B29 lysine of insulin (green) using a DBCO-sulfo-NHS crosslinker (not drawn to scale). **c**, Agarose gel electrophoresis of the scaffold strand and of insulin NanoRods with 0 (NR), 1 (NR-1), 2 (NR-2), 4 (NR-4), 7 (NR-7) and 15 (NR-15) insulin molecules. The image is representative of three independent experiments. **d**, Analysis of the indicated insulin NanoRods by dynamic light scattering. **e**, Schematic of DNA-PAINT used to image INS-DNA bound to the NanoRods. Distribution of the occupancy of insulin on the indicated insulin NanoRods. The values presented under the images correspond to mean ± s.d. Scale bar, 50 nm. Source data

**Extended Data Fig. 2:**
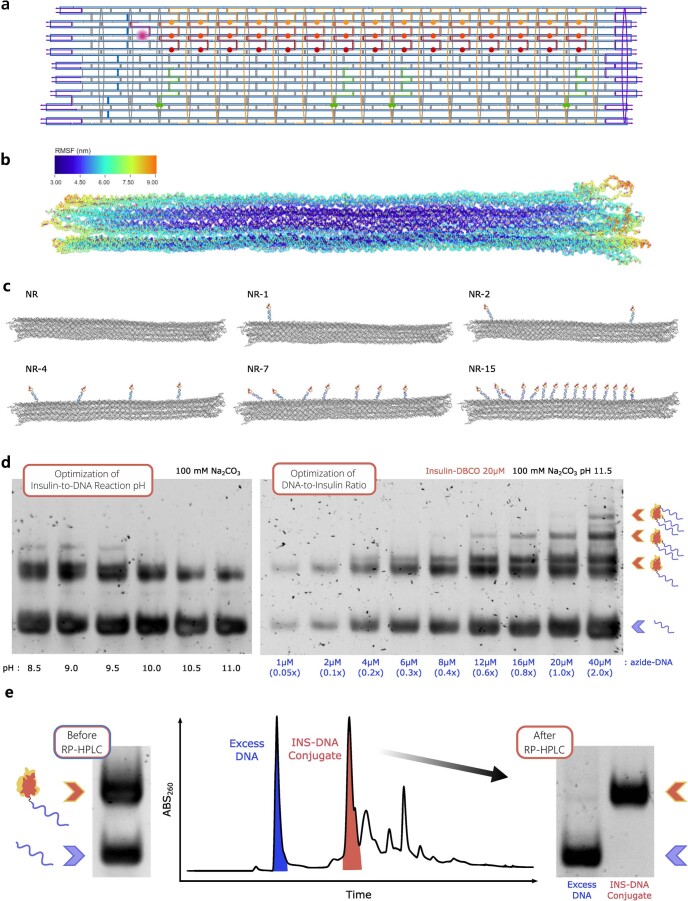
Characterization of insulin-DNA conjugates and insulin NanoRods. **a**, The 18-helix tube DNA origami design (referred to as NanoRod) showing staple positions, as visualized with caDNAno software. Colour coding was as follows: green = biotin staples, orange/red = possible insulin-DNA attachment sites, purple = staples at end of rod. **b**, Rigidity analysis of the NanoRod structure using the oxDNA coarse-grained modelling software (available at https://oxDNA.org) and visualized via the browser-based online tool oxView. Rigidity analysis showed some fluctuations at the ends of the rod but a stable solid core. RMSF: root mean square fluctuations. **c**, Snapshots from the oxDNA simulations for the indicated insulin NanoRods. Insulin peptides were not possible to included in the DNA-only simulations but insulin peptides (red) were drawn in afterwards to illustrate their scale. **d**, Optimization of conjugation conditions of insulin to DNA. Images of native polyacrylamide gels stained with SYBR Gold to visualize azide-DNA and insulin-DNA conjugates. The pH of the reaction and the ratio of DBCO-modified insulin to azide-DNA were optimized in order to obtain a single DNA strand conjugated to the insulin. In these experiments DBCO-modified insulin (insulin-DBCO) was kept at a constant concentration of 20 µM and the azide-DNA was used in a 1:1 ratio for the pH optimization experiments or used in a range of 1-40 µM in the optimization experiments of insulin-DBCO/azide-DNA ratio. Optimizations were performed once. Gels were acquired with different exposures. Values in brackets indicate azide-DNA: insulin-DBCO ratio. **e**, The insulin-DNA conjugation protocol resulted in an excess of unconjugated azide-DNA (bottom band, left gel, blue peak in HPLC trace) which required purification via RP-HPLC for separation from the insulin-DNA (INS-DNA) conjugate (top band, left gel, red peak HPLC trace). HPLC purification was able to entirely remove free azide-DNA (right gel). Analysis of purified conjugates was carried for every production batch. Source data

**Extended Data Fig. 3:**
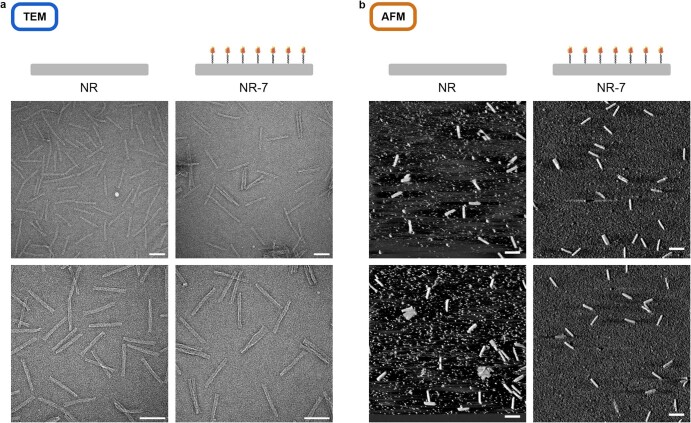
TEM and AFM imaging of NanoRods and insulin NanoRods. **a**, Representative TEM images of NR and NR-7 nanostructures. Scale bars 100 nm. **b**, Representative AFM phase images of NR and NR-7 nanostructures. Scale bars 200 nm. Images representative of 2 independent experiments.

The DNA origami technique^22^ generates user-defined nanoscale shapes that can be engineered to present single-stranded DNA (ssDNA) oligonucleotides at defined locations, addressable by complementary ssDNA strands conjugated to biomolecules of interest, thereby assembling them into nanopatterns^10,23,24^. We selected an 18-helix DNA origami tube design with a triangular cross section^10^ (Fig. 2a and Extended Data Fig. 2a,b), referred to as a NanoRod here, with a nominal length of 140 nm (Fig. 2a), which is in the same length scale as the dimensions of IR nanoclusters. We designed a row of potential protruding ssDNA sites along the length of the NanoRod, to be addressed by insulin–DNA conjugates (INS-DNA) (Fig. 2a,b and Extended Data Fig. 2c–e). The NanoRod has a high persistence length and the resulting insulin–DNA origami nanostructures, which we termed as insulin NanoRods, deliver well-defined insulin spatial stimuli to cells in a one-dimensional manner, and with radial symmetry of receptor binding at an IR nanocluster population level. This enables a reduction in nano-organization stimuli variable space compared with two-dimensional designs. We made a palette of insulin NanoRods with 0 (NR), 1 (NR-1), 2 (NR-2), 4 (NR-4), 7 (NR-7) or 15 (NR-15) insulin molecules spaced along the length of the NanoRod (Extended Data Fig. 2c). The positions of the insulin molecules closest to the edges were the same for all the insulin NanoRods in this series, to exclude differences in receptor binding related to ligand proximity to the edges^25^; this resulted in a constant span of 105 nm between the edge insulin molecules. Agarose gel electrophoresis analysis showed that increasing the incorporation of INS-DNA in the nanostructures resulted in increasing shifts in band migration (Fig. 2c). In addition, dynamic light scattering showed that the different insulin NanoRods had similar particle sizes (Fig. 2d), indicating that the introduction of insulin molecules does not induce aggregation. Transmission electron microscopy (TEM) and atomic force microscopy (AFM) imaging confirmed the integrity of the insulin NanoRods, but the insulin molecules were not detectable due to their small size (Extended Data Fig. 3). To analyse insulin incorporation in the nanostructures, we performed DNA-PAINT imaging (Fig. 2e). This confirmed that the INS-DNA localized to the intended binding sites, with average numbers of insulin molecules per nanostructure slightly below those defined by the designs, particularly for those with higher valencies (Fig. 2e), as previously reported^12,26^.

### Insulin valency controls the residence time at IRs

**Fig. 3:**
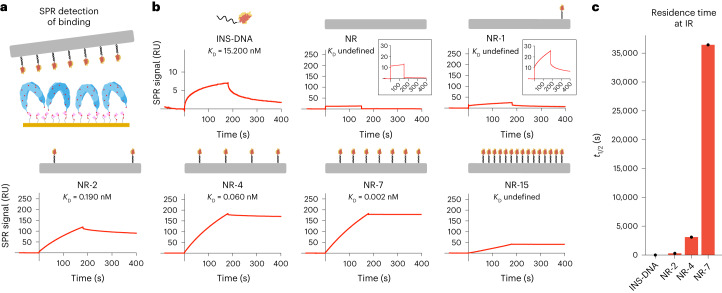
Valency of insulin on NanoRods determines the residence time of IR binding. **a**, Schematic of the SPR assay: biotinylated ECD-IR was immobilized onto a streptavidin-coated SPR surface followed by incubation with INS-DNA or insulin NanoRods. **b**, SPR traces showing the binding of INS-DNA and of the indicated insulin NanoRods to the ECD-IR. *K*_D_ was calculated from the curves obtained from INS-DNA, NR-2, NR-4 and NR-7. RU, resonance units. **c**, Residence time (*t*_1/2_) of the insulin NanoRods and INS-DNA on ECD-IR, calculated from the SPR binding curves. The values shown are from one representative experiment out of two independent experiments. Source data

**Extended Data Fig. 4:**
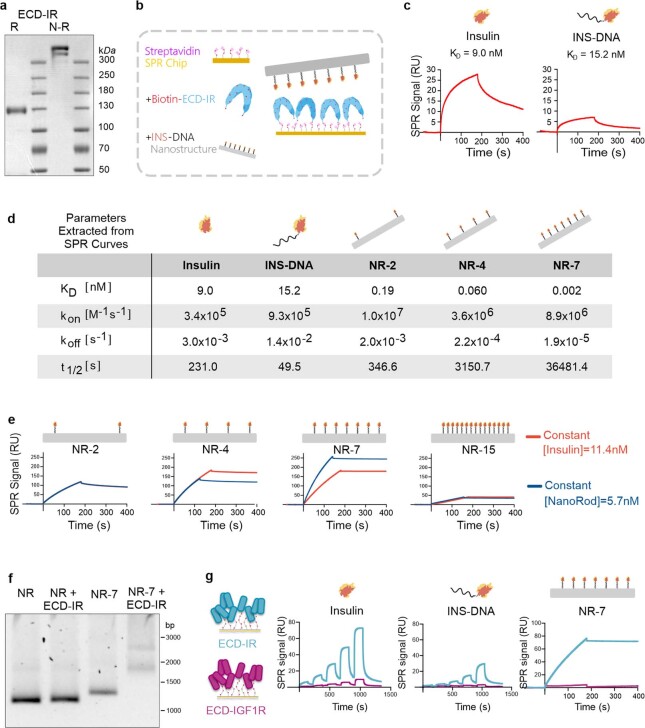
Characterization of insulin and insulin NanoRod binding to IR by SPR and gel shift assays. **a**, Coomasie gel of recombinant extracellular domain of human IR (ECD-IR) in non-reducing (N-R) or reducing (R) conditions. In reducing conditions ECD-IR runs at approximately the molecular weight predicted for the monomeric protein. In non-reducing conditions proteins run at a higher molecular weight, indicating that the ECD-IR proteins in our assays are present as dimeric protein complexes. **b**, Schematic of the components of the SPR set-up. **c**, SPR traces of unmodified insulin (INS) and INS-DNA conjugates (the latter also shown in Fig. 3b). **d**, Parameters extracted from SPR curves in Fig. 3, for structures where values could be determined, and from the SPR curve in panel (c) for unmodified insulin. We were not able to determine parameters for the NR, NR-1, and NR-15 because the SPR sensorgrams, both in association and dissociation phase, could not be fitted by the available models. **e**, SPR experiments were done both at constant insulin concentration (11.4 nM, red curves also shown in Fig. 3b), or at constant NanoRod concentration (5.7 nM, blue curves). Similar overall trends were observed in the two sets of data. **f**, Agarose gel stained for DNA of gel shift binding assays showing that NR-7, but not NR, bind to ECD-IR. **a,f**, Images representative of 2 independent experiments. **g**, SPR sensorgrams of insulin, INS-DNA and NR-7 binding to sensor chips with immobilized ECD-IR (blue curve) or ECD-IGF1R (magenta curve). Binding of insulin and INS-DNA was tested using a range of insulin concentrations (6.2, 18.5, 55.6, 166.7, and 500 nM). Binding of NR-7 was tested at a single concentration of the nanostructure (11.4 nM insulin). Source data

To analyse the binding kinetics of insulin NanoRods to the IR, we performed surface plasmon resonance (SPR) using dimerized IR extracellular domains (ECD-IR) (Extended Data Fig. 4a), immobilized on the sensor chip (Fig. 3a and Extended Data Fig. 4b). Insulin binding to ECD-IR had a dissociation constant *K*_D_ in the low nanomolar range, in agreement with the literature^5^, whereas the *K*_D_ value of INS-DNA was slightly increased (Extended Data Fig. 4c,d). The obtained sensograms showed stark differences in the binding kinetics between the nanostructures, when either insulin (Fig. 3b and Extended Data Fig. 4d) or NanoRod (Extended Data Fig. 4e) concentrations were kept constant. In particular, NR-7 showed a substantial decrease in the kinetic dissociation rate constant (*k*_off_) to the immobilized ECD-IR (Extended Data Fig. 4d), which translated into a residence time approximately three orders of magnitude higher than that of INS-DNA (Fig. 3c and Extended Data Fig. 4d). *K*_D_ could not be defined for NR-15 due to poor curve fitting, but the sensogram showed a flat dissociation curve, indicative of a low *k*_off_ value. Gel shift assays confirmed that NR-7, but not NR, was able to bind ECD-IR (Extended Data Fig. 4f), and SPR analysis revealed that NR-7, similar to insulin, showed minimal binding to the extracellular domain of IGF1R—a membrane receptor closely related to IR (Extended Data Fig. 4g). Together, these results show that the binding of insulin NanoRods to ECD-IR is specific, insulin-mediated and determined by insulin valency.

### Insulin valency and spacing determine pathway activation

**Fig. 4:**
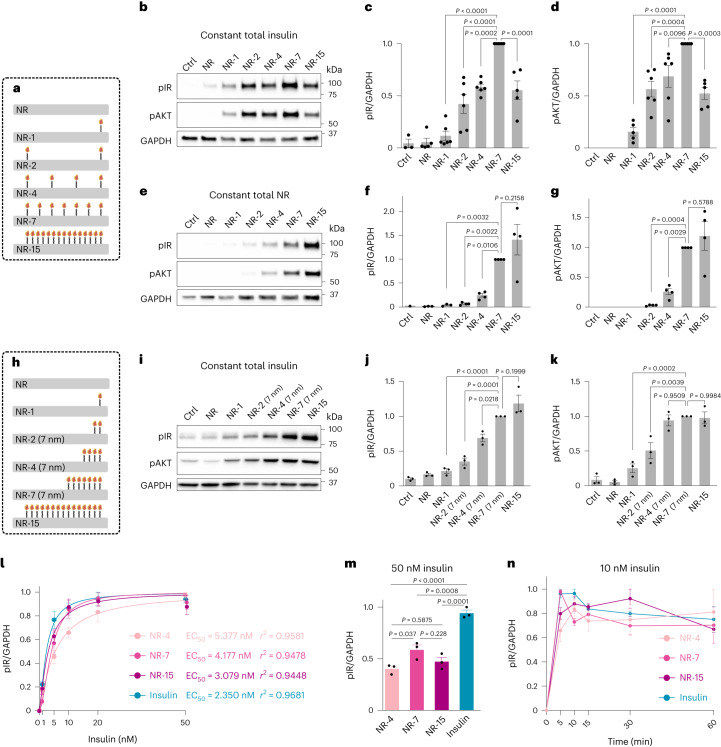
Valency and spacing of insulin on NanoRods determine IR pathway activation. **a**, Schematic of the NanoRods used in experiments (**b**–**g** and **l**–**n**). **b**–**d**, Western blot analysis (**b**) and the quantification of phosphorylated IR (pIR) (**c**) and phosphorylated AKT (pAKT) (**d**) levels in adipocytes treated with medium as controls (Ctrl) or with the indicated insulin NanoRods for 10 min. The total insulin concentration was kept constant at 10 nM. The values are presented as mean ± standard error of the mean (s.e.m.); *n* = 5 (NR-15) or *n* = 6 (remaining conditions) biologically independent samples. **e**–**g**, Western blot analysis (**e**) and the quantification of pIR (**f**) and pAKT (**g**) levels of adipocytes treated with medium (Ctrl) or with the indicated insulin NanoRods for 10 min. The total concentration of NanoRods was kept constant at 1 nM. The values are presented as mean ± s.e.m.; *n* = 4 biologically independent samples. **h**, Schematic of the NanoRods used in experiments (**i**–**k**). **i**–**k**, Western blot analysis (**i**) and the quantification of pIR (**j**) and pAKT (**k**) levels of adipocytes treated with medium (Ctrl) or with the indicated insulin NanoRods for 10 min. The total insulin concentration was kept constant at 10 nM. The values are presented as mean ± s.e.m.; *n* = 3 biologically independent samples. **c**,**d**,**f**,**g**,**j**,**k**, *P* values determined by one-way ANOVA followed by Dunnett’s multiple comparisons test. **l**, Quantification of pIR levels in adipocytes treated with increasing total insulin concentrations of NR-4, NR-7, NR-15 and unmodified insulin for 10 min. Data are plotted as normalized intensities relative to their highest and lowest values, for each condition. **m**, Quantification of pIR levels in adipocytes treated with 50 nM total insulin of NR-4, NR-7, NR-15 and unmodified insulin for 10 min. *P* values determined by one-way ANOVA followed by Dunnett’s multiple comparisons test. **n**, Quantification of pIR levels in adipocytes treated with 10 nM total insulin of NR-4, NR-7, NR-15 and unmodified insulin for 5, 10, 15, 30 and 60 min. Data are plotted as normalized intensities relative to their highest and lowest values, for each condition. The values in **l**–**n** are presented as mean ± s.e.m. for *n* = 3 biologically independent samples. The unmodified insulin used was purified similar to the NanoRods. Source data

**Extended Data Fig. 5:**
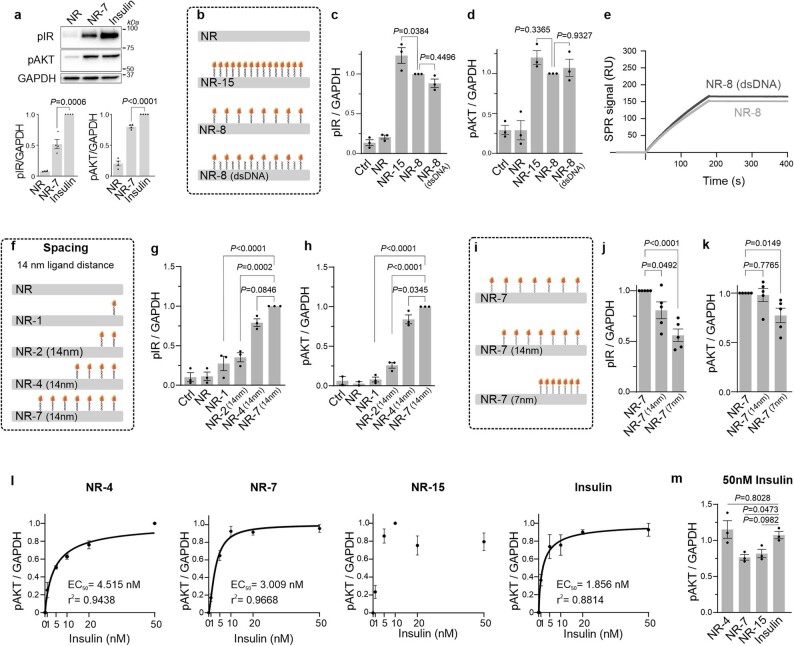
Characterization of the effects of insulin spacing and valency on IR-pathway activation. **a**, Western blot analysis and quantification of pIR and pAKT levels of differentiated adipocytes treated with NR, NR-7 and insulin for 10 min at 10 nM of total insulin concentration. Values presented as mean ± SEM; n = 4 biologically independent samples. *P* values, unpaired two-tailed *t* test. **b**, Schematic representation of structures used in (**c-e**). **c,d**, Western blot quantifications of pIR (**c**) and pAKT (**d**) levels of differentiated adipocytes treated with medium as controls (Ctrl) or with indicated insulin NanoRods for 10 min. The total concentration of NanoRods was kept constant at 10 nM. Values presented as mean ± SEM; n = 3 biologically independent samples. *P* values, one-way ANOVA followed by Dunnet’s multiple comparisons test. **e**, SPR sensorgrams of the binding of NR-8 and NR-8(dsDNA) structures to the ECD-IR on the SPR chip. RU, resonance units. **f**, Schematic representation of structures used in (**g,h**) **g,h**, Western blot quantifications of pIR (**g**) and pAKT (**h**) levels of differentiated adipocytes treated with medium (Ctrl) or with indicated insulin NanoRods for 10 min. The total insulin concentration was kept constant at 10 nM. Values presented as mean ± SEM; n = 3 biologically independent samples. *P* values, one-way ANOVA followed by Dunnet’s multiple comparisons test. **i**, Schematic representation of structures used in (**j,k**). **j,k**, Western blot quantifications of pIR (**j**) and pAKT (**k**) levels of differentiated adipocytes treated with indicated insulin NanoRods for 10 min. The total insulin concentration was kept constant at 10 nM. Values presented as mean ± SEM; n = 5 biologically independent samples. *P* values, unpaired two-tailed *t* test. **l,m**, Quantification of pAKT levels in adipocytes treated with NR-4, NR-7, NR-15, and unmodified insulin for 10 min at increasing concentrations of total insulin. Unmodified insulin used in these experiments was purified similarly to the insulin NanoRods. Data plotted as normalized intensities relative to their highest and lowest values, for each of the conditions (l). Values presented as mean ± SEM, n = 3 biologically independent samples. *P* values, one-way ANOVA followed by Dunnet’s multiple comparisons test. Source data

To analyse the effects of insulin valency on IR activation, we treated cultured adipocytes with insulin NanoRods of various valencies for 10 min, at a constant total insulin concentration, and analysed the phosphorylation levels of IR as well as the downstream protein AKT by western blot (Fig. 4a,b). All the insulin NanoRods activated the IR, but importantly, the activation levels varied greatly. NR-7 exhibited the strongest activation of the IR pathway, with both IR (Fig. 4c) and AKT (Fig. 4d) phosphorylation peaking with this insulin NanoRod configuration. However, NR-7 showed lower phosphorylation levels than unmodified insulin (Extended Data Fig. 5a), consistent with previous studies showing that ligand presentation by DNA origami nanostructures is often associated with decreased ligand binding to cells^25^. In these experiments, NR-15, which had the highest valency but the lowest NanoRod concentration, resulted in lower levels of IR activation than NR-7. Interestingly, when the concentration of NanoRods was kept constant (Fig. 4e–g), instead of the total concentration of insulin, NR-15 still did not result in significantly increased receptor activation compared with NR-7; NR-15 and NR-7 show similar IR and AKT phosphorylation levels (Fig. 4f,g). To address whether the NR-15 design is associated with steric hindrance due to the 7 nm spacing of the protruding dsDNA oligonucleotides, we made NanoRods where every other position in NR-15 had an empty protruding dsDNA oligonucleotide that did not present an insulin molecule (NR-8dsDNA) (Extended Data Fig. 5b). The levels of IR activation induced by NR-8dsDNA and by the corresponding insulin NanoRods without the empty protruding dsDNA oligos (NR-8) were similar and slightly lower than those of NR-15, at a constant NanoRod concentration (Extended Data Fig. 5c). Further, there were no differences in AKT phosphorylation between NR-15, NR-8 and NR-8dsDNA (Extended Data Fig. 5d). SPR assays also indicated similar binding properties of NR-8dsDNA and NR-8 to immobilized ECD-IR (Extended Data Fig. 5e). Together, these results indicate that the lower IR-activating capacity per presented insulin exhibited by NR-15 cannot be attributed to steric hindrance effects.

To investigate the effects of insulin spacing on IR activation, we designed a series of insulin NanoRods with different valencies but with a constant spacing of 7 nm (NR-2 (7 nm), NR-4 (7 nm) and NR-7 (7 nm)) (Fig. 4h) or 14 nm (NR-2 (14 nm), NR-4 (14 nm) and NR-7 (14 nm)) (Extended Data Fig. 5f). For both spacings, increasing the insulin valency resulted in increased IR phosphorylation, reaching saturation at NR-7 (7 nm) (Fig. 4i,j) and NR-4 (14 nm) (Extended Data Fig. 5g), respectively. AKT phosphorylation levels also increased with valency, up to NR-4 (7 nm) (Fig. 4i,k) and NR-7 (14 nm) (Extended Data Fig. 5h). Importantly, treatment with NR-7 (7 nm) resulted in lower IR and AKT phosphorylation than NR-7 (Extended Data Fig. 5i–k), confirming that the 7 nm spacing was suboptimal for promoting IR activation. Together, these results show that both insulin valency and spacing control the IR activation levels.

**Extended Data Fig. 6:**
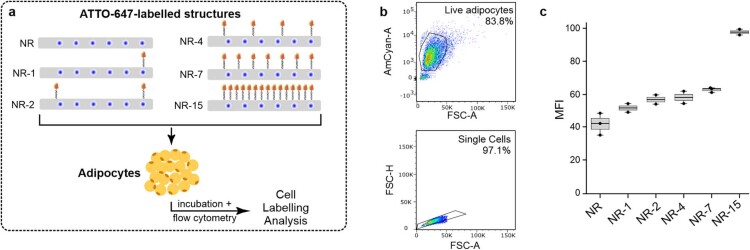
Characterization of cell labelling by insulin NanoRods. **a**, Schematic summary of the flow cytometry assay. NanoRod structures were labelled with six ATTO-647 fluorophores. Dissociated adipocytes were incubated for 10 min with 10 nM NanoRod structures before flow cytometry analysis. **b**, Dot plot graphs from flow cytometry analysis representing gates for live and single cell adipocytes. **c**, Box plots of mean fluorescent intensity (MFI) for adipocytes treated with indicated structures. Data was normalized to untreated cells. Boxplot data: the center line defines median, the box indicates 95% CI, and the whiskers indicate SD. n = 3 (NR and NR-7 conditions) or n = 2 (remaining conditions) biologically independent samples. Source data

**Extended Data Fig. 7:**
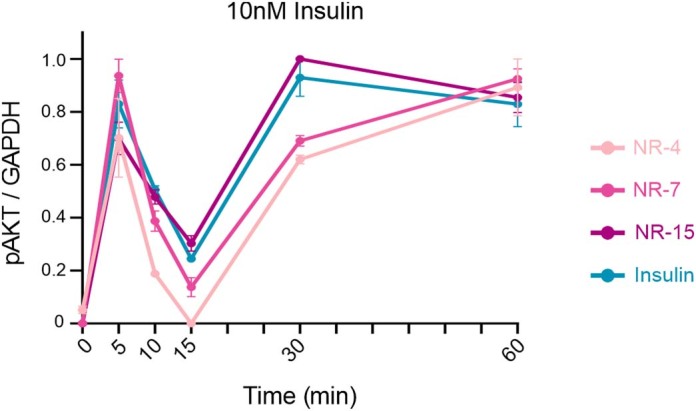
Characterization of IR-pathway activation over time. Quantification of pAKT levels in adipocytes treated with 10 nM total insulin of NR-4, NR-7, NR-15, and unmodified insulin for 5, 10, 15, 30 and 60 min. Data plotted as normalized intensities relative to their highest and lowest values, for each of the conditions. Values presented as mean ± SEM, n = 3 biologically independent samples. Source data

To determine the half-maximal effective concentration (EC50) of insulin NanoRods compared with unmodified insulin, we treated cells with increasing insulin concentrations of NR-4, NR-7, NR-15 and unmodified insulin. We analysed the phosphorylation levels of IR (Fig. 4l) and AKT (Extended Data Fig. 5l) and plotted the band intensities of the western blot relative to their highest and lowest values, for each of the concentration series. We found that unmodified insulin had the lowest EC50 value and that the EC50 value of insulin NanoRods decreased with increasing valency (Fig. 4l). To directly compare the maximum levels of receptor activation, cells were treated with 50 nM of insulin or with NanoRods at 50 nM insulin concentration (Fig. 4m and Extended Data Fig. 5m). Unmodified insulin was associated with higher maximum IR phosphorylation levels than insulin NanoRods. Interestingly, NR-15 showed the lowest EC50 but not the highest maximum IR phosphorylation amongst the tested NanoRod configurations. Further, flow cytometry analysis of cells treated with insulin NanoRods revealed that cell labelling increased with insulin valency, with NR-15 showing the highest levels, at a constant NanoRod concentration (Extended Data Fig. 6). Together, these results suggest that NR-15 has the highest cell-binding capacity amongst the tested insulin NanoRods, whereas NR-7 leads to the highest levels of receptor activation. To analyse the dynamics of pathway activation, cells were treated with insulin NanoRods or with unmodified insulin for 0, 5, 10, 15, 30 and 60 min. We did not observe marked differences in the normalized IR activation dynamics amongst the insulin NanoRods and insulin, with IR phosphorylation increasing at 5 min and staying largely stable for the time points analysed (Fig. 4n), whereas AKT phosphorylation showed an initial increase followed by a minimum at 15 min (Extended Data Fig. 7). This indicates that the effects of insulin NanoRods on the observed IR activation cannot be attributed to differences in the dynamics of IR activation.

**Fig. 5:**
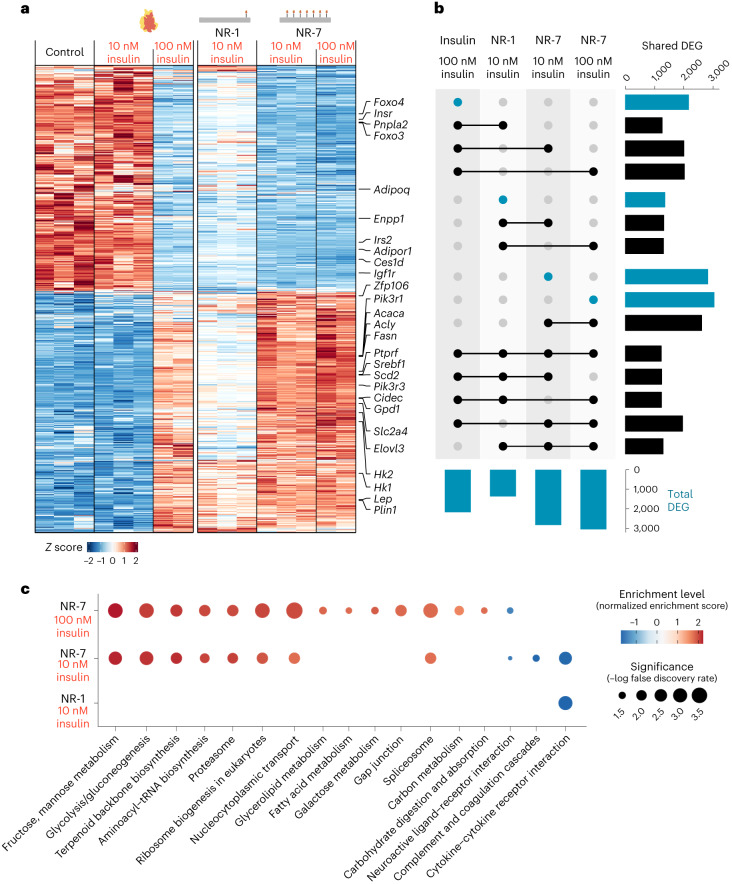
Valency of insulin on NanoRods modulates transcriptional responses. **a**, Heat map of DEG (*P* ≤ 0.001; log_2_fold change, ±0.58) in adipocyte cultures treated with medium (Control), insulin at 10 or 100 nM, NR-1 at 10 nM of insulin and NR-7 at 10 nM or 100 nM of insulin for 4 h. **b**, UpSet plot visualizing the total number (blue bars) and shared (black bars) DEG across all the conditions for which DEG were detected. **c**, GSEA with false-discovery rate adjustment for KEGG pathways enriched (*P* ≤ 0.05) in cells treated with NR-1 at 10 nM insulin or NR-7 at 10 and 100 nM insulin. The highlighted categories indicate pathways associated with insulin metabolism. INS denotes insulin. Source data

**Extended Data Fig. 8:**
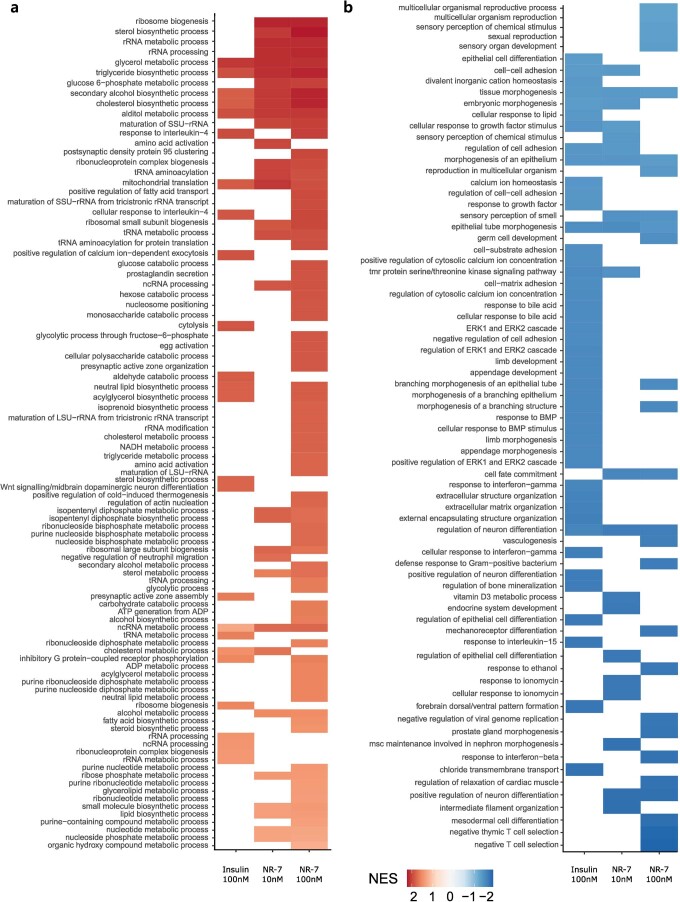
Gene set enrichment analysis (GSEA) of GO-Terms. GSEA was generated from a ranked list of DE genes from INS (100 nM), NR-7 (10 nM and 100 nM) treated adipocytes, and the degree of enrichment is indicated by the normalized enrichment score (NES) with positive (**a**) or negative (**b**) enrichment scores. The GO-Terms were considered significantly enriched with an FDR adjusted p-value cutoff at 0.1. No GO-Terms were significantly enriched for NR-1 (10 nM) treated adipocytes.

Next, we compared the effects of multivalent insulin presentation by NR-7 with the monovalent presentation of NR-1 on the transcriptional responses of adipocytes using mRNA sequencing. Insulin signalling in adipocytes promotes glucose uptake, glycolysis, *de novo* lipogenesis, triacylglyceride storage and glycogen synthesis, and represses lipolysis and the release of free fatty acids. Cells were treated with unmodified insulin or with NR-7 or NR-1 and analysed after 4 h. We identified approximately 2,200 differentially expressed genes (DEG) in cultures treated with unmodified insulin at 100 nM compared with control cultures (Supplementary Table 1). In contrast, few DEG were detected in cells treated with unmodified insulin at 10 nM (Fig. 5a). Interestingly, treatment with NR-7 or NR-1, at 10 nM of insulin, resulted in a considerable number of DEG. Further, NR-7 treatment resulted in a higher number of DEG (2,841 genes) than NR-1 treatment (1,372 genes) (Fig. 5a,b and Supplementary Table 1). Additionally, out of the identified insulin-regulated genes, 91.5% were also identified in NR-7-treated cultures, whereas only 57.5% were identified in NR-1-treated cultures (Fig. 5b). However, 91.5% of NR-1-regulated genes were also regulated by unmodified insulin (Fig. 5b). It is noteworthy that increasing the insulin concentration from 10 to 100 nM in the NR-7 treatment led to only slight transcriptional changes (Fig. 5a,b), suggesting that the insulin-mediated transcriptional response of NR-7 reaches the maximum at 10 nM of insulin—a concentration where NR-1 or unmodified insulin showed little effect. Gene set enrichment analysis (GSEA) for Gene Ontology terms (Extended Data Fig. 8) and KEGG pathways (Fig. 5c and Supplementary Table 2) of the identified DEG revealed that upregulated genes showed significant enrichment for pathways associated with insulin signalling in adipocytes such as fatty acid, glycerol/glycerolipid and glucose metabolism. SREBF and FOXO transcription factors regulate transcriptional networks important for orchestrating insulin metabolic responses in adipocytes with reciprocal roles; SREBF promotes the expression of genes involved in lipogenesis and triacylglyceride storage, whereas FOXO induces genes associated with lipolysis. NR-7 and NR-1 induced the expression of *Srbf1*, with higher fold change for NR-7 (Fig. 5a and Supplementary Table 1). Further, NR-7 induced higher fold changes than NR-1 in the expression of SREBF1 target genes related to *de novo* lipogenesis (for example, *Fasn*, *Acly*, *Acaca* and *Scd2*) and lipid droplet organization/storage (*Plin1*) (Fig. 5a and Supplementary Table 2). In addition, the expression of the lipase *Pnpla2* and adiponectin (*Adipoq*), which are target genes of FOXO1 and inversely correlated with insulin stimulation, was downregulated by NR-7 but not NR-1 (Fig. 5a and Supplementary Table 1). Together, these data show that multivalent insulin presentation results in stronger IR pathway activation and transcriptional responses than monovalent insulin.

### Multivalent insulin lowers free glucose levels in zebrafish

**Fig. 6:**
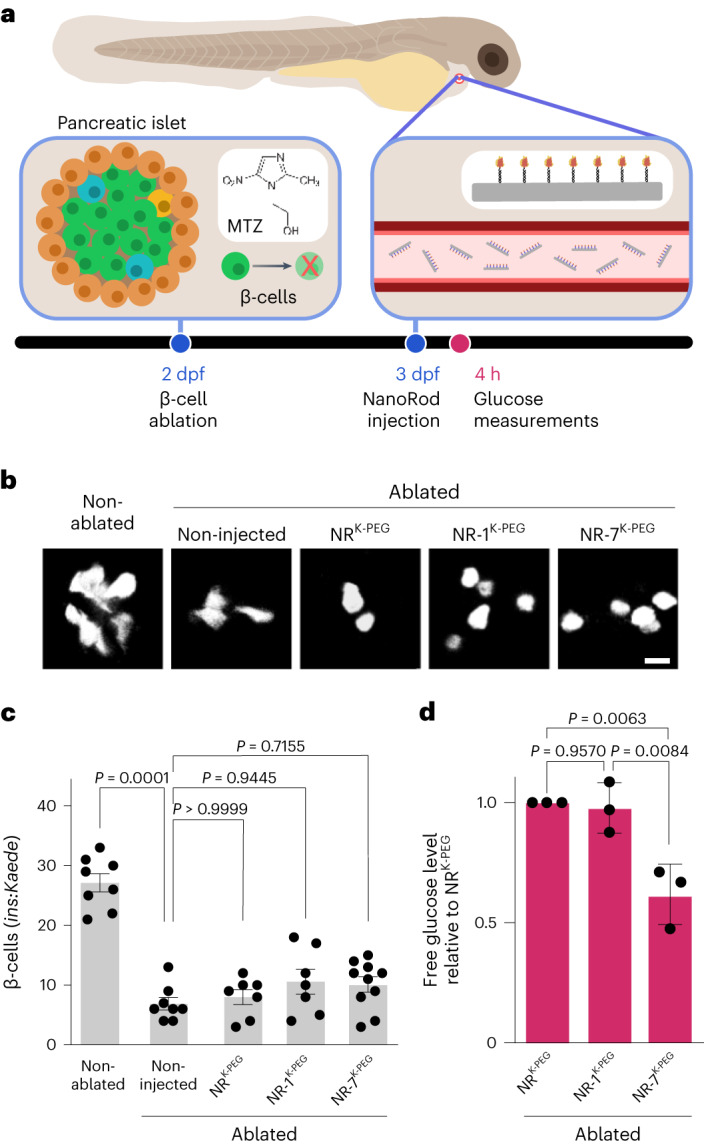
Valency of insulin on NanoRods determines their capacity to lower free glucose in β-cell-ablated zebrafish larvae. **a**, Schematic of the zebrafish model, which expresses the enzyme nitroreductase (NTR) under the control of the insulin promoter and converts the MTZ compound into a toxic byproduct that ablates β-cells. Larvae were treated with MTZ at 2 dpf for 24 h. Double-transgenic larvae, *Tg*(*ins*:*CFP*-*NTR*);*Tg*(*ins*:*Kaede*), were used to visualize β-cells with the fluorescent protein Kaede. NR^K-PEG^, NR-1^K-PEG^ or NR-7^K-PEG^ was intravenously injected into larvae at 3 dpf and the measurements of free glucose levels were taken 4 hpi. **b**, Confocal microscopy imaging of the Kaede fluorescent protein expressed in pancreatic β-cells in the indicated conditions. Scale bar, 10 µm. **c**, Bar plots of the quantifications of Kaede^+^ β-cells. Two independent experiments were performed; one representative experiment is shown where each dot corresponds to a larva. The values are presented as mean ± s.e.m. *n* = 8 (non-ablated, ablated non-injected), *n* = 7 (NR^K-PEG^, NR-1^K-PEG^), *n* = 10 (NR-7^K-PEG^). The *P* values are determined by a Kruskal–Wallis test with Dunn’s multiple comparisons test. **d**, Bar plots of free glucose levels in NR-1^K-PEG^- and NR-7^K-PEG^-treated larvae relative to NR^K-PEG^-treated larvae. The values are presented as mean ± s.d. *n* = 3 independent experiments (Extended Data Fig. 9e). The *P* values are determined by one-way ANOVA with Tukey’s multiple comparisons test. Source data

**Extended Data Fig. 9:**
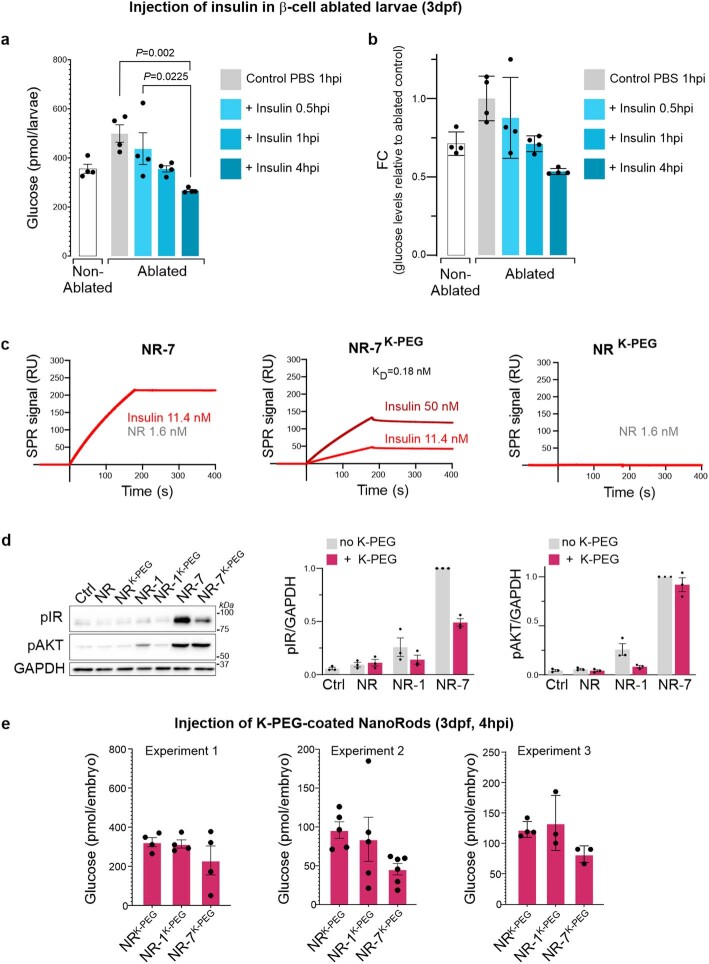
Analysis of free glucose levels in β-cell-ablated zebrafish larvae and characterization of oligolysine-PEG coated insulin NanoRods. **a**, Measurement of free glucose levels in β-cell ablated zebrafish larvae at various time points post-injection of 2 nl of 100 nM insulin. Non-ablated larvae and β-cell-ablated larvae injected with PBS were used as controls. Data presented as mean ± SD. n = 4 biologically independent samples. Each dot corresponds to measurements from a pool of 3-6 larvae. *P* values, one-way ANOVA with Turkey´s multiple comparison test. hpi, hours post-injection. **b**, Replotting of free glucose levels data from panel (**a**) relative to ablated (control) condition. Values presented as mean ± SD. n = 4 biologically independent samples. **c**, SPR traces showing the binding of indicated insulin NanoRods to the ECD-IR on the SPR chip. K_D_ for oligolysine-PEG-coated NR-7 (NR-7^K-PEG^) was obtained from sensorgrams using 50 nM concentration of total insulin. No binding was detected for oligolysine-PEG-coated NR (NR^K-PEG^) structures, which were used as a control indicating that coating does not result in non-specific binding. RU, resonance units. **d**, Western blot analysis and quantification of pIR and pAKT levels of adipocytes treated with uncoated (NR, NR-1, NR-7) or coated (NR^K-PEG^, NR-1^K-PEG^, NR-7^K-PEG^) structures. Values in bar plots presented as mean ± SEM; n = 3 biologically independent samples. **e**, Measurement of free glucose levels in β-cell ablated zebrafish larvae 4 hours post injection of NR ^K-PEG^, NR-1^K-PEG^, and NR-7^K-PEG^. Data presented as mean ± SEM. For Experiment 1, n = 4; Experiment 2, n = 5 (NR ^K-PEG^, NR-1^K-PEG^) and n = 6 (NR-7^K-PEG^); Experiment 3, n = 4 (NR ^K-PEG^) and n = 3 (NR-1^K-PEG^, NR-7^K-PEG^) biologically independent samples. Data corresponds to measurements from three separate experiments conducted on three separate occasions and was used to generate data presented in Fig. 6d. Each dot corresponds to a measurement from a pool of 3-6 larvae. Source data

To investigate the functional effects of multivalent (NR-7) versus monovalent (NR-1) delivery of insulin in vivo, we used the zebrafish (*Danio rerio*) model *Tg*(*ins*:*CFP*-*NTR*) engineered for conditionally targeted β-cell ablation by metronidazole (MTZ) treatment^27^. Additionally, we used the *Tg*(*ins*:*CFP*-*NTR*);*Tg*(*ins*:*Kaede*) transgenic line, where the expression of the fluorescent protein Kaede is under the control of the insulin promoter, to visualize β-cells^28^ (Fig. 6a). We induced β-cell ablation by MTZ treatment at two days post-fertilization (2 dpf), resulting in reduced numbers of Kaede^+^ β-cells (Fig. 6b,c) and increased free glucose levels (Extended Data Fig. 9a,b) at 3 dpf. To determine the time required to observe a reduction in free glucose levels after β-cell ablation, we injected insulin at 3 dpf and analysed free glucose levels 0.5, 1.0 and 4.0 h post-injection (hpi). We observed a consistent normalization of free glucose levels at 4 hpi (Extended Data Fig. 9a,b) and used this time point for subsequent analysis. To promote the stability and bioavailability of DNA nanostructures in vivo, insulin NanoRods were coated with oligolysine-PEG (K-PEG), which increases the resistance to nuclease digestion^29,30^. We confirmed that coated NR-7 (NR-7^K-PEG^) was able to bind ECD-IR by SPR, but with lower affinity compared with uncoated NR-7, and that the K-PEG coating did not lead to unspecific binding (Extended Data Fig. 9c). Further, NR-1^K-PEG^ and NR-7^K-PEG^ induced IR phosphorylation, although at lower levels compared with their uncoated counterparts (Extended Data Fig. 9d). Of note, the relationship between phosphorylation levels induced by the multivalent and monovalent insulin NanoRods was maintained (Extended Data Fig. 9d). We injected NR-7^K-PEG^ and NR-1^K-PEG^, at a constant insulin concentration, as well as NR^K-PEG^ as a control, in ablated zebrafish larvae at 3 dpf. We observed that the nanostructures did not significantly affect the numbers of Kaede^+^ β-cells (Fig. 6b,c). Importantly, treatment with NR-7^K-PEG^ significantly decreased the free glucose levels to below those of the control (NR^K-PEG^) whereas NR-1^K-PEG^ structures did not show a significant effect (Fig. 6d and Extended Data Fig. 9e). Therefore, multivalent insulin presentation facilitates the reduction in free glucose levels in this zebrafish model.

## Conclusions

The development of insulin therapeutics has focused on tuning individual insulin–insulin or insulin–IR-binding interactions without taking into account the overarching organization of IRs at the cell membrane. Interestingly, the exposure of cells to bivalent antibodies to the IR, but not the corresponding monovalent Fab fragments, has long been known to elicit various insulin-like responses^31^, suggesting that multivalent receptor activation can regulate cellular responses. Here we revisit this concept and demonstrate that IRs form nanoclusters at the cell membrane and that the valency and spatial organization of engineered insulin nanoclusters control the residence time at the IR, receptor activation, transcriptional responses and in vivo functionality.

**Extended Data Fig. 10:**
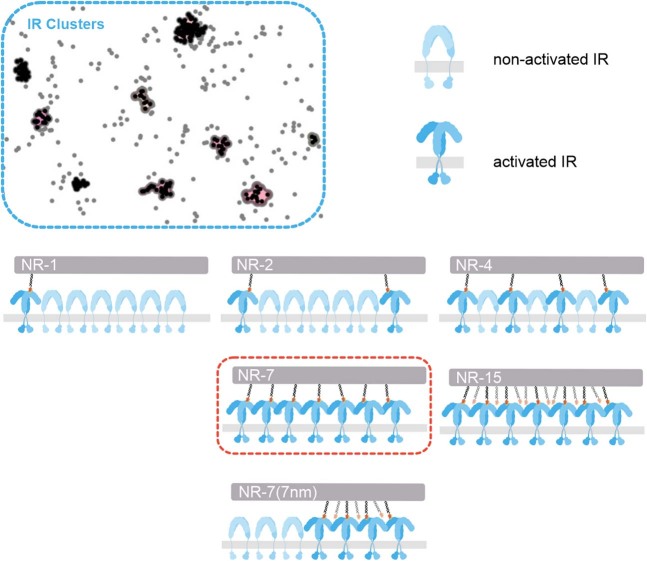
Insulin receptor clustering at the cell membrane enables avidity effects between insulin receptor and insulin NanoRods. DNA-PAINT super-resolution imaging revealed the presence of IR clustering at the cell membrane. NR-7 presents insulin molecules with a nominal average spacing of 17 nm and IR dimers are approximately 12 nm wide. Therefore, these nanostructures may offer the closest match between the distribution of insulin ligands in the nanostructures and IRs at the cell membrane. Further, increasing the number of insulin molecules per nanostructure, and therefore decreasing their spacing, as in NR-15, may result in “unused” insulin molecules.

Amongst the nanostructures tested, NR-7 showed the highest IR-activating capacity per presented insulin. We propose that this is due to a combination of valency and insulin spacing in NR-7, as decreasing either parameter resulted in lower IR activation. We hypothesize that our results are consistent with a model where NR-7 provides, amongst the structures tested, the highest potential to elicit avidity effects between the multivalent insulin nanocluster and the IR nanoclusters at the cell membrane that are conducive to IR activation (Extended Data Fig. 10).

We envision that this work unlocks the possibility of exploiting differences in IR spatial organization to achieve specific insulin targeting. Of particular interest are potential differences in IR spatial organization between tissues and between normal and insulin-resistant states. Additionally, as knowledge advances on the composition of membrane IR nanoclusters, multicomponent nanostructures containing insulin and other ligands or binders that simultaneously target IR and other membrane receptors may be used to tailor cellular responses to insulin stimulation.

Together, our results suggest that rather than changing the insulin concentration or altering the chemistry of the peptide, simply modulating the valency and geometry of insulin nanoclusters can control IR-mediated responses and may provide a starting point for the development of new drugs for the treatment of diabetes.

## Methods

### Cell culture

Brown adipocytes were differentiated from an immortalized brown preadipocyte mouse cell line kindly provided by B. Spiegelman (Harvard University) as previously described^19^. Preadipocytes were maintained and expanded at low confluence (<50%) in a growth medium (Dulbecco’s modified Eagle’s medium, high glucose (Gibco), supplemented with 20% foetal bovine serum (Sigma-Aldrich), 20 mM HEPES (Sigma-Aldrich) and 100 U ml^−1^ penicillin–streptomycin (Gibco)). For differentiation, preadipocytes were washed twice with phosphate-buffered solution (PBS) (Gibco, pH 7.4, 1×), dissociated with TrypLE Express, and seeded at a density of approximately 23,000 cells cm^−2^ in the growth medium. The medium was changed after 24 h to a differentiation medium (Dulbecco’s modified Eagle’s medium, high glucose, supplemented with 10% foetal bovine serum, 20 nM insulin, 1 nM triiodothyronine and 100 U ml^−1^ penicillin–streptomycin) and the cells were cultured for 2 days. Cell differentiation was then induced by the addition of an induction medium (differentiation media supplemented with 0.125 mM indomethacin, 0.5 μM dexamethasone and 0.5 mM isobutyl methylxanthine) for 2 days, after which the medium was changed to the differentiation medium for two additional days. Differentiated adipocytes were washed once with a starvation medium (Dulbecco’s modified Eagle’s medium, low glucose (Gibco), supplemented with glucose to a final concentration of 8 mM, 0.5% bovine serum albumin (Sigma-Aldrich) and 100 U ml^−1^ penicillin–streptomycin) and incubated for 2 h in the starvation medium before treatment with insulin NanoRods or NanoRods diluted in the starvation medium for the times indicated in the main text. Following treatment, the cells were washed once in PBS and harvested for the isolation of protein for immunoblots or RNA for gene expression. For IR analysis by DNA-PAINT, preadipocytes were differentiated as described above with the following modifications. After treatment with the induction medium, the cells were dissociated with TrypLE Express and seeded in the differentiation medium in μ-Slide 18 Well Glass Bottom wells (ibidi). After 2 days, the cells were incubated with the starvation medium for 2 h followed by treatment with 10 nM insulin in the starvation medium for 10 min. In control experiments, the addition of insulin was omitted. Before treatment, differentiated adipocytes were visually analysed to assess the cell surface density and to evaluate the differentiation of cells, and then randomly allocated to the treatment and control groups.

The cells were maintained, expanded and differentiated in a humidified atmosphere containing 5% CO_2_ at 37 °C.

### DNA-PAINT of IR

Differentiated adipocytes were fixed for 12 min at room temperature (RT) with pre-warmed 4% paraformaldehyde in PBS, washed three times with PBS, blocked for 90 min at RT with a blocking solution (3.0% foetal bovine serum/0.1% Triton X-100 in PBS) and incubated with rabbit anti-IR β antibody (Cell Signaling (4B8); 1:300 dilution in the blocking solution) for 2 days at 4 °C. The cells were then washed three times with PBS and incubated with the nanobody FluoTag-XM-QC anti-rabbit IgG (Massive Photonics) diluted 1:200 in a blocking buffer (Massive Photonics) for 1 h at RT. Following three washes with PBS, the cells were incubated with 80 nm gold nanoparticles (Sigma-Aldrich; 1:5 dilution in the blocking buffer) for 10 min. The cells were washed once with PBS and incubated with 5 nM Cy3b-labelled strands (Massive Photonics) diluted in an image buffer (Massive Photonics).

Imaging was carried out on a Nikon ECLIPSE Ti-E microscope with a Perfect Focus system (Nikon Instruments), applying an objective-type total internal reflection fluorescence configuration using an iLAS2 circular total internal reflection fluorescence module (Gataca Systems) with an oil-immersion 1.49-numerical-aperture CFI Plan Apo total internal reflection fluorescence ×100 objective (Nikon Instruments) equipped with ×1.5 auxiliary Optovar magnification corresponding to a final pixel size of 87 nm. The laser used was an OBIS 561 nm LS 150 mW (Coherent) with custom iLas input beam expansion optics (Cairn) optimized for reduced field super-resolution imaging. The fluorescent light beam was passed first through a filter cube (89901, Chroma Technology) containing an excitation quadband filter, a quadband dichroic filter and a quadband emission filter (ZET405/488/561/640x, ZET405/488/561/640bs and ZET405/488/561/640m, Chroma Technology). Fluorescence light was then spectrally filtered with an emission filter (ET595/50m, Chroma Technology) and imaged on an iXon Ultra 888 electron-multiplying charge-coupled device camera (Andor). Micro-Manager software v. 1.4 was used to acquire 12,000 frames with 10 MHz readout frame, 130 ms exposure and no electron multiplication gain. A total of ten cells from three independent experiments were imaged in each condition (Supplementary Note and Supplementary Fig. 1).

### INS-DNA production and purification

Insulin (Merck, 1 mg ml^−1^) was reacted with dibenzocyclooctyne-sulfo-*N*-hydroxysuccinimidyl ester (DBCO-sulfo-NHS, Click Chemistry Tools; 690 µM) in 100 mM Na_2_CO_3_ buffer (pH 11.5) for 20 min at RT. The reaction was then quenched for 5 min through the addition of Tris base (Sigma-Aldrich, 100 mM). The solution was washed three times with 400 µl of 100 mM Na_2_CO_3_ using Amicon Ultra 0.5 ml centrifugal filter units with a 3 kDa cut-off membrane (Merck). At each washing step, the columns were spun for 10 min at 14,000×*g*. After the final washing step, insulin–DBCO (690 µM) was mixed with azide-modified DNA (Biomers, 35 µM; Supplementary Table 3) in 100 mM Na_2_CO_3_ and left to react for 3 h at RT. The reaction was quenched by adding NaN_3_ (Sigma-Aldrich, 6.9 mM). The samples were run on a native polyacrylamide gel (6% 19:1 polyacrylamide in 1× TAE, 20 min, 200 V, TAE running buffer) and stained with SYBR Gold (Thermo Fisher) according to the manufacturer’s instructions, for confirming INS-DNA formation. Imaging was performed using an ImageQuant LAS 4000 gel imager. The insulin–DBCO-sulfo-NHS conjugation protocol was optimized to promote the binding of the ssDNA oligo to lysine-29 of the B chain (B29 lysine) compared with the amine groups at the *N* terminus of insulin A and B chains. The pKa value of the B29 amine is higher than those of the amines of the two *N* termini (11.2 versus 8.6 and 6.8). At high pH, the NHS group of the crosslinker is predicted to preferentially react with the most basic amine group, which is the B29 lysine amine^32^. Therefore, the pH reaction conditions were optimized to promote a single INS-DNA product.

INS-DNA reaction mixes were purified by a reverse-phase high-performance liquid chromatography C_18_ column (Agilent Poroshell 120 EC-C_18_) on an Amersham Pharmacia Biotech ÄKTA Ettan LC. Buffer A (50 mM triethylamine acetate) and buffer B (90% acetonitrile and 10% buffer A) were used in a gradient profile, in which the percent of buffer B was increased from 30% to 50% over 20 min. Fractions were collected and spun on a vacuum concentrator (Thermo Scientific SpeedVac Savant DNA 120) for 30 min at high heat to remove the volatile components of the high-performance liquid chromatography buffers. Selected peaks were buffer exchanged into PBS using Amicon Ultra 0.5 ml centrifugal filter units with a 3 kDa cut-off membrane (Merck), by spinning three times for 10 min at 14,000×*g* and washing each time with 400 µl PBS. Samples of purified fractions were run on a native polyacrylamide gel and stained with SYBR Gold (Thermo Fisher) to visualize the purified INS-DNA. The final purity of conjugates was analysed for every preparation via three methods: comparison of silver staining band intensities (Pierce Silver Stain Kit) on sodium dodecyl sulfate–polyacrylamide gel electrophoresis (Invitrogen, 4–12% Bolt gel) against insulin standards (Merck), comparison of SYBR Gold band intensity on native polyacrylamide gel electrophoresis against DNA standards (Integrated DNA Technologies) and through the Qubit ssDNA Assay Kit (Qubit 4 Fluorimeter, Invitrogen). Final concentrations of INS-DNA were calculated based on Qubit ssDNA measurements. Purified INS-DNA was frozen and stored at −20 °C until further use.

### Production of NanoRods and insulin NanoRods

Origami structures were prepared by mixing scaffold plasmid DNA (p7560, Tilibit, 10 nM) with the appropriate staple strands (Integrated DNA Technologies, 100 nM) (Supplementary Tables 4–9) in a folding buffer (5.0 mM Tris at pH 8.5 (Sigma-Aldrich), 1.0 mM EDTA (Panreac AppliChem) and 12.5 mM MgCl_2_ (Sigma-Aldrich)). The mix was then placed in a thermocycler (MJ Research PTC-225 Gradient Thermal Cycler) and annealed by heating to 80.0 °C for 5 min, cooling to 60.0 °C at 1.0 °C per min over 20 min and then slowly cooling to 20.0 °C at 0.5 °C per min. Excess staples were removed using Amicon Ultra 0.5 ml centrifugal filter units with a 100 kDa cut-off membrane (Merck) by spinning five times for 2 min at 14,000×*g* and washing each time with 400 µl of the folding buffer. The concentration of the purified structure was determined by measuring the DNA absorbance at 260 nm (Thermo Scientific NanoDrop 2000). Purified INS-DNA was then added in 3× stoichiometric excess to available extended strand-binding sites on the NanoRod structure and annealed in a thermocycler by heating to 37.0 °C for 1 h, cooling to 22.0 °C at 0.1 °C per min, incubating at 22.0 °C for 14 h and cooling to 4.0 °C at 0.1 °C per min. Unbound INS-DNA was removed using Amicon Ultra 0.5 ml centrifugal filter units with a 100 kDa cut-off membrane (Merck), spinning five times for 2 min at 14,000×*g* and washing each time with 400 µl of PBS + 10 mM MgCl_2_. Insulin NanoRods were stored at 4 °C until further use.

### Agarose gel electrophoresis

NanoRod structures were analysed by running samples on agarose gels on ice for 4 h at 70 V. Gels were composed of 2% agarose (Thermo Scientific TopVision Agarose) in 0.5× TBE buffer (Panreac AppliChem) plus 10 mM MgCl_2_ (Sigma-Aldrich) and 1× SYBR Safe DNA stain (Invitrogen). Imaging was performed using an ImageQuant LAS 4000 gel imager.

### Dynamic light scattering

NanoRod and insulin NanoRod samples were prepared in PBS + 10 mM MgCl_2_, syringe filtered using 0.1 µm membranes (Merck) and analysed on a Zetasizer Ultra instrument (Malvern Panalytical). Three measurements were taken at 25 °C in a low-volume cell (ZSU1002) and then averaged.

### oxDNA simulation of the NanoRod

NR, NR-1, NR-2, NR-4, NR-7 and NR-15 structures were analysed using oxDNA coarse-grained modelling (https://oxdna.org/). NanoRod structures with dsDNA strands extending from the sites of insulin incorporation were created using vHelix and converted to the oxDNA format using the tacoxDNA web site (http://tacoxdna.sissa.it/). Structures were submitted to the oxDNA.org web server for simulation at 37 °C, with 1 as the salt concentration, 1 × 10^8^ time steps with a d*t* value of 0.0001, and a preliminary relaxation step with the default parameters. Simulations were visualized and videos were made using the oxView tool (https://oxdna.org/).

### Negative-staining TEM

Purified NanoRods (10 nM) were dispensed on a glow-discharged carbon-supported copper TEM grid (TEM-CF200CU50, Thermo Fisher Scientific) and incubated for 60 s before removing the solution. The grids were then stained for 10 s with 5 µl of 2% w/v uranyl formate, which was subsequently removed. The staining procedure was repeated seven times and the TEM grids were air dried for 30 min before imaging. Imaging was performed on a Talos 120C G2 (120 kV, Ceta-D detector) at ×92,000 for near-field views. Raw images were processed using ImageJ software (v1.53).

### AFM

NanoRod structures were imaged on a disc of mica fastened with epoxy adhesive to the centre of a microscope slide and enclosed by a plastic ring attached to the slide using Reprorubber. Nanostructures were diluted to 1 nM in TE-Mg buffer (5 mM Tris base, 1 mM EDTA, 10 mM MgCl_2_, pH 8.0) and 10 µl was pipetted onto freshly cleaved mica. After 30 s, 4 µl of 5 mM NiSO_4_ was added and incubated for a further 4.5 min. The surface was then rinsed with 1.0 ml of 0.1 µm-filtered TE-Mg buffer after which 1.5 ml of filtered TE-Mg buffer was added to the mica disc for imaging. Imaging was performed in liquid using a JPK Instruments NanoWizard 3 Ultra atomic force microscope with a Bruker AC40 cantilever in the a.c. mode.

### DNA-PAINT of insulin NanoRod nanostructures

The µ-Slide 18 Well Glass Bottom wells (ibidi) were cleaned with isopropanol and dried with N_2_. The wells were incubated with 1 mg ml^−1^ Biotin bovine serum albumin (Thermo Fisher) in buffer A (10 mM Tris-HCl, 100 mM NaCl and 0.05% (v/v) Tween 20 at pH 8.0) for 5 min at RT, washed three times with buffer A and incubated with 0.5 mg ml^−1^ neutravidin (Thermo Fisher) in buffer A for 5 min at RT. Wells were then washed three times with buffer A followed by three washes with buffer B (5 mM Tris-HCl, 10 mM MgCl_2_, 1 mM EDTA and 0.05% (v/v) Tween 20 at pH 8.0). Then, 500 pM NanoRods incorporating four biotin-labelled DNA strands (Extended Data Fig. 2a), with INS-DNA containing a 9-nucleotide PAINT docking sequence (DS1; Supplementary Table 3), was added to each well for 5 min. The wells were washed three times with buffer B. Then, 1 nM of Atto-647N imager strand (IS1; Supplementary Table 10) in buffer B supplemented with oxygen scavengers (protocatechuic acid (Sigma), protocatechuate 3,4-dioxygenase (Sigma) and Trolox (Sigma)) was added to each well. For dual-exchange PAINT, three docking sequences (DS2; Supplementary Table 10) were added on both ends of the NanoRods. Samples were prepared as previously described, with wells being washed ten times with buffer B between each imaging acquisition. Each imaging acquisition was done with a different Atto-647N imager strand (IS1 and IS2; Supplementary Table 10). Micro-Manager software was used to acquire 9,000 frames with 10 MHz readout frame, 200 ms exposure and no electron multiplication gain (Supplementary Note and Supplementary Figs. 2 and 3).

### SPR

A Biacore T200 instrument (Cytiva) was used to perform the SPR experiments and data were acquired using Biacore T200 system control software v. 2.01. Biotinylated ECD-IR (Nordic BioSite) was immobilized on a streptavidin Sensor Chip SA (Cytiva). HBS-P+ buffer (Cytiva) was used as the running buffer. After the immobilization of ECD-IR, a stabilization time of 15 min was introduced to reach a stable baseline. NR-1, NR-2, NR-4, NR-7, NR-15, NR-8 and NR-8dsDNA were injected at 11.4 nM concentration of insulin in the running buffer. Insulin and INS-DNA were injected at 55 nM, the minimum concentration to obtain a binding curve that could be analysed. NR was injected as a negative control at a concentration equal to the highest concentration of NanoRod used within insulin NanoRod injections (NR-1 = 11.4 nM). Alternatively, NR-2, NR-4, NR-7 and NR-15 were injected at 5.7 nM concentration of nanostructure (Extended Data Fig. 4e), and NR-7^K-PEG^ was also injected a 50 nM concentration of insulin (Extended Data Fig. 9c). The injection of each sample was performed using an association phase of 180 s and a dissociation phase of 300 s. The dissociation equilibrium constant (*K*_D_), association rate constant (*k*_on_) and dissociation rate constant (*k*_off_) were determined using the BIAevaluation 3.0 software. The *t*_1/2_ values, which define the residence time, were determined using the formula ln2/*k*_off_. To compare the binding of NR-7 structures between IR and IGF1R, ECD-IR and ECD-IGF1R proteins (Nordic BioSite) were immobilized on two different flow cells of a CM5 sensor chips via amine coupling reactions, according to the manufacturer’s instructions. The binding of insulin and INS–DNA was tested by injecting different concentrations of insulin (6.2, 18.5, 55.6, 166.7 and 500.0 nM) in the running buffer (HBS-P+) in the single-cycle kinetic mode, using an association phase of 140 s and a dissociation phase of 300 s. Binding of NR-7 was tested by injecting a single concentration of structure (11.4 nM of insulin) using an association phase of 180 s and a dissociation phase of 300 s.

### Coomassie gel

Here 0.5 µg of recombinant ECD-IR (Nordic BioSite) was resuspended in Laemmli sample buffer (Bio-Rad). For reducing conditions, 2-mercaptoethanol was added to a final concentration of 2.5%. The samples were denaturated at 80 °C for 10 min, resolved by sodium dodecyl sulfate–polyacrylamide gel electrophoresis and stained with GelCode Blue safe protein stain (Thermo Fisher Scientific).

### Gel shift assay

NanoRods (NR and NR-7) at 20 nM were incubated with 300 nM recombinant extracellular domain of human IR (Nordic BioSite) or with PBS for 30 min at 4 °C. The samples were then run on a 2% agarose gel and stained with SYBR Safe.

### Immunoblotting

The cells were washed with PBS, lysed in radioimmunoprecipitation assay buffer (Sigma-Aldrich) supplemented with protease and phosphatase inhibitor cocktail (Thermo Fisher Scientific) and incubated on ice with shaking for 30 min. The lysate was cleared by centrifugation (20,000×*g* for 20 min at 4 °C) and the protein lysates were quantified using the Bradford protein assay (Bio-Rad). Protein lysates were resuspended in the Laemmli sample buffer (Bio-Rad) containing 2.5% of 2-mercaptoethanol, denaturated at 80 °C for 10 min, resolved by sodium dodecyl sulfate–polyacrylamide gel electrophoresis and transferred onto polyvinylidene fluoride membranes. Membranes were incubated 1 h in a blocking solution (Tris-buffered saline with 0.1% Tween 20 (TBST) and 5.0% non-fat dry milk), followed by overnight incubation at 4 °C with primary antibodies against phospho-IR beta/IGF1R beta (CST 3024, 1:1,000), phospho-AKT-S473 (CST 4058, 1:1,000) or GAPDH (Invitrogen PA1-987, 1:5,000). After three washes with TBST, the membranes were incubated with horseradish-peroxidase-conjugated secondary antibodies (Invitrogen, 31460, 1:5,000) for 1 h at RT. Detection of horseradish peroxidase was performed by chemiluminescent substrate Immobilon Forte on a ChemiDoc Imaging System (Bio-Rad). Band densiometry was performed using the ImageJ software.

### Flow cytometry

Differentiated, serum-starved adipocytes were prepared as described above. Adipocytes were subsequently washed twice with Hanks’ balanced salt solution (HBSS) and dissociated in collagenase D solution (1.5 U ml^−1^ collagenase D (Roche) and 10 mM CaCl_2_ in HBSS) for 20 min at 37 °C. The cells were resuspended in HBSS, filtered through a 35 µm HBSS-equilibrated cell strainer (BD Biosciences), pelleted at 300×*g* for 5 min and resuspended in a staining buffer (1× PBS and 1% bovine serum albumin). The dead cells were labelled with LIVE/DEAD Fixable Yellow Dead Cell Stain Kit (Thermo Fisher Scientific) according to the manufacturer’s instructions, and subsequently, the cell suspension was pelleted at 300×*g* for 5 min and resuspended in the staining buffer. Here ~100,000 cells were incubated with 10 nM ATTO-647-labelled NanoRod structures in a final volume of 100 µl for 10 min at 37 °C. The cells incubated without the NanoRod structures were used as the untreated control. The cells were then washed twice with the staining buffer by centrifugation at 300×*g* for 5 min. Flow cytometry measurements were performed on a BD FACSCANTO II with BD FACSDIVA software v. 9.0 (BD Biosciences). Live adipocyte cells were initially identified by gating cells on FSC-A versus AmCyan-A, followed by gating on FSC-A versus FSC-H to detect singlets. The acquired data were analysed using FlowJo 10.7.1 software (BD Biosciences). The geometric mean of the fluorescence intensity after normalization to the untreated control was used to define the degree of cell labelling by the NanoRods.

### RNA-seq library preparation and sequencing

The total RNA was isolated from differentiated adipocytes using Quick-RNA Microprep Plus Kit (Zymo Research), and 500 ng of purified RNA was used for mRNA-seq library preparation using TruSeq RNA Library Prep Kit v2 according to the manufacturer’s low-sample protocol. Library quantification was performed using the QuantiFluor dsDNA system (Promega) according to the manufacturer’s multiwell plate protocol on a Varioskan LUX multimode microplate reader (Thermo Fisher). The library size and quality were assessed using Bioanalyser 2100 and High Sensitivity DNA Kit (Agilent). Libraries were denatured and diluted using NextSeq standard normalization protocol (Illumina), and sequencing was performed using single-end reads (1 × 75 bp) with NextSeq 500/550 High Output Kit v. 2.5 (75 cycles) on a NextSeq 550 platform (Illumina).

### RNA-seq quantification, DEG analysis and GSEA

Sequencing reads were mapped against a reference transcriptome of *Mus musculus* protein-coding transcript sequences (release M29, GRCm39; https://www.gencodegenes.org/mouse/) and quantified using Salmon 1.7.0 (ref. ^33^). Count tables were generated using the tximport package^34^ and lists of DEG were obtained using the DESeq2 package (v. 1.34.0)^35^, where only genes with adjusted *P* values equal to or below 0.001 and a log_2_fold change cut-off at ±0.58 were considered for further analysis. Heat maps and UpSet plots were generated using ComplexHeatmap (v. 2.10.0)^36^. GSEA for biological processes with both Gene Ontology terms and KEGG pathways was performed using a ranked list of genes as input to clusterProfiler (v. 4.2.2)^37^ and a significance of false-discovery-rate-adjusted *P* values below 0.10 and 0.05, respectively.

### Zebrafish microinjections and free glucose quantification

NanoRod and insulin NanoRods were mixed with oligolysine-PEG (K_10_-PEG_5__K_, Alamanda Polymers) at a 1:1 ratio between the amines of lysines in K_10_-PEG_5K_ and the phosphates in DNA^30^, and incubated at RT for 30 min before a microinjection of 2 nl of the sample into each zebrafish larva. The samples for injection were prepared at a final concentration of 100 nM of structures for the coated NanoRod samples and at a final concentration of 100 nM insulin for the coated insulin NanoRod samples (corresponding to 100.0 and 14.3 nM of NanoRods for NR-1^K-PEG^ and NR-7^K-PEG^, respectively). Injection of 1 nl of human insulin at 100 nM concentration in zebrafish larvae has been shown to induce a decrease in free glucose levels and transcriptional changes consistent with insulin signalling^38^. We, therefore, injected 2 nl of 100 nM insulin concentration in our assays to evaluate the insulin-mediated effects on free glucose levels. Since the total blood volume for a 2 dpf zebrafish is 60–89 nl (ref. ^39^), the estimated concentration of the injected insulin in our assays would be around 2–3 nM. In these assays, we were also limited in the amount of sample injected, with higher injection levels (3 and 4 nl) resulting in poor larvae survival.

The maintenance and crossing of zebrafish (*D. rerio*) lines were conducted in compliance with Swedish legislation on animal welfare regulations approved by Stockholms djurförsöksetiska nämnd. Since for β-cell ablation and free glucose assay experiments only animals younger than 5 days were used, no ethical permit was required according to 2010/63/EU. Zebrafish transgenic lines used have been previously described, namely, *Tg*(*ins*:*CFP*-*NTR*)^*s892*^ (ref. ^27^) and *Tg*(*ins*:*Kaede*)^*s949*^ (ref. ^28^).

Ablation of β-cells was performed in two-day-old *Tg*(*ins*:*CFP*-*NTR*) and *Tg*(*ins*:*CFP*-*NTR*);*Tg*(*ins*:*Kaede*) embryos by treatment with 10 mM MTZ (Sigma-Aldrich) diluted in 1% DMSO (VWR) in an egg water solution (E3) supplemented with 0.2 mM 1‐phenyl‐2‐thiourea (PTU, Acros Organics) for 24 h. Following β-cell ablation, three-day-old *Tg*(*ins*:*CFP*-*NTR*) larvae (72 hpf) were anaesthetized in 0.01% tricaine and injected with 2 nl of 1× PBS, unmodified insulin or coated NanoRod/insulin NanoRods into the common cardinal vein (duct of Cuvier)^40^. Phenol red (Sigma-Aldrich) to a final concentration of 0.1% was added to the PBS, insulin or coated insulin NanoRod samples to aid in the visualization of the microinjection process and the determination of successfully injected larvae. Zebrafish larvae were randomly assigned to the treatment groups. Free glucose levels were measured as described elsewhere^41^ using a fluorescence-based enzymatic kit (BioVision). Groups of three to six injected larvae were used per condition/replicate.

### Confocal imaging

*Tg*(*ins*:*CFP*-*NTR*);*Tg*(*ins*:*Kaede*)-ablated larvae were collected 24 h after ablation treatment, anaesthetized and injected following the previously stated protocol and fixed in 4% paraformaldehyde solution before analysing the β-cell numbers by confocal imaging. The confocal images were acquired with a Leica TCS SP8 microscope and the LAS X software (v. 3.5.5.19976). The primary pancreatic islets of the β-cell-ablated *Tg*(*ins*:*CFP*-*NTR*);*Tg*(*ins*:*Kaede*) larvae were scanned with a ×40 water-immersion objective and the *z* stacks were analysed using Fiji software (v1.53). All the displayed images were acquired from the same experiment and their contrast values were adjusted for visualization purposes. The quantification of β-cells was performed on original unmodified images.

### Statistical analysis

No statistical methods were used to predetermine the sample sizes, but the sample sizes were similar to those reported in previous publications^28,38,42^. Cell culture samples and animals were randomly assigned to the control and treatment groups. Data collection and analysis were not performed blind to the conditions of the experiments. Individual data points are plotted for most graphs. Sample size (*n*) of the number of experimental biological repeats and the statistical methods used are indicated in the corresponding figure legends. Datasets were tested for Gaussian distribution followed by the appropriate statistical test. Statistical analysis and graphical representation of the data were processed with GraphPad Prism 9.4.0. Two-tailed Mann–Whitney test was performed to compare the cluster properties between the control and insulin-treated cells. For western blot quantifications, one-way analysis of variance (ANOVA) followed by Dunnett’s multiple comparisons test was carried out. For the quantification of β-cells, the Kruskal–Wallis test followed by Dunn’s multiple comparisons test was carried out. The analysis of free glucose values was performed using one-way ANOVA with Tukey’s multiple comparisons test.

### Reporting summary

Further information on research design is available in the Nature Portfolio Reporting Summary linked to this article.

## Online content

Any methods, additional references, Nature Portfolio reporting summaries, source data, extended data, supplementary information, acknowledgements, peer review information; details of author contributions and competing interests; and statements of data and code availability are available at 10.1038/s41565-023-01507-y.

### Supplementary information

Supplementary Information Supplementary Figs. 1–3 and Note.

Reporting Summary

Supplementary Data 1 Supplementary Tables 1–10.

### Source data

Source Data Fig. 1 Statistical source data.

Source Data Fig. 2 Statistical source data.

Source Data Fig. 2 Unprocessed gels.

Source Data Fig. 3 Statistical source data.

Source Data Fig. 4 Statistical source data.

Source Data Fig. 4 Unprocessed western blots.

Source Data Fig. 5 Statistical source data.

Source Data Fig. 6 Statistical source data.

Source Data Extended Data Fig. 1 Statistical source data.

Source Data Extended Data Fig. 2 Unprocessed gels.

Source Data Extended Data Fig. 4 Statistical source data.

Source Data Extended Data Fig. 4 Unprocessed gels.

Source Data Extended Data Fig. 5 Statistical source data.

Source Data Extended Data Fig. 5 Unprocessed western blots.

Source Data Extended Data Fig. 6 Statistical source data.

Source Data Extended Data Fig. 7 Statistical source data.

Source Data Extended Data Fig. 9 Statistical source data.

Source Data Extended Data Fig. 9 Unprocessed western blots.

## Data Availability

RNA-seq data are available via the ArrayExpress database at https://www.ebi.ac.uk/biostudies/arrayexpress under accession number E-MTAB-12160. Reference *Mus musculus* protein-coding transcriptome release M29 (GRCm39) (https://www.gencodegenes.org/mouse/). Source data are provided with this paper.

## Code availability

The code used for the analysis of DNA-PAINT of IRs and RNA-seq data is available via GitHub at https://github.com/TeixeiraLab/Spratt-et-al.
