# Supplementary material for: Multivalent insulin receptor activation using insulin–DNA origami nanostructures
Source: Nat Nanotechnol. 2023 Oct 9;19(2):237–45. doi: 10.1038/s41565-023-01507-y (PMC10873203; doi:10.1038/s41565-023-01507-y)
Supplement: Supplementary file 1 — Supplementary Figs. 1–3 and Note. [file 41565_2023_1507_MOESM1_ESM.pdf]

nature nanotechnology

Article

https://doi.org/10.1038/s41565-023-01507-y

Multivalent insulin receptor activation using insulin–DNA origami nanostructures

In the format provided by the authors and unedited

Contents

Supplementary Notes **2**

DNA-PAINT of insulin receptors at the adipocyte cell membrane 2

Cluster analysis of insulin receptors at the adipocyte cell membrane 2

DNA-PAINT of insulin NanoRods 2

qPAINT analysis of insulin NanoRods 3

Supplementary Figures **4**

Supplementary Fig. 1. Processing and analysis steps in DNA-PAINT experiments 4

Supplementary Fig. 2. Characterization of the number of insulin molecules bound to the insulin NanoRods by DNA-PAINT 5

Supplementary Fig. 3. DNA-PAINT images of insulin NanoRods 6

References **7**

Supplementary Notes

DNA-PAINT of insulin receptors at the adipocyte cell membrane

Picasso Localize was used to detect localizations by using a minimum net gradient of 2000 and the MLE algorithm with 0.001 and 1000 as criterion convergence and maximum number of iterations, respectively. Picasso Filter was used to filter out localizations based on their localization precision error and Gaussian fitting (Supplementary Fig. 1a). Picasso Render was used to correct sample drift by first using redundant cross-correlation (RCC) and then by using gold nanoparticles as fiducial markers (Supplementary Fig. 1b). For RCC, a segmentation value of 1000 was used. Gold nanoparticles were then picked and the feature "undrift using picked" was used. Localizations were then linked to remove blinking events appearing in multiple consecutive frames. The feature "link localizations" was used at a maximum distance (pixel) equal to the localization precision error and a maximum transient dark frame of 15. Localizations were then exported as .csv file for further processing. Thunderstorm was used to open the localization file and export a 20x20 µm region of interest as a .csv file.

Cluster analysis of insulin receptors at the adipocyte cell membrane

Localizations were analyzed using the trained neural network model 87B144[1] using the 1000 nearest neighbours as input (Supplementary Fig. 1c). Briefly, using the Localization file data, a JSON file was generated with the zzz_MakeJSON.py script. The JSON and the localization files were then used to calculate the distances between each point and its nearby neighbour point using the script 1.0_Data_Preparation.py. The script 4.0_Evaluation_With_ClusterClub.py was used to evaluate the prepared data using model 87B144. The script 5.0_Info_by_ROIs.py was finally used to extract cluster information for further analysis. To discriminate clusters from single receptors, histograms of the numbers of localizations per cluster were plotted and fitted with a multiple Gaussian to obtain the mean number of localizations per receptor[2] (Extended Data Fig. 1a). Statistical analysis was performed using a two-tailed non-parametric Mann-Whitney test in GraphPad prism software 9.3.1 with $P < 0.05$ for rejecting the null hypothesis.

A custom-written Python code was used to calculate distances between neighboring clusters. The nearest edge distances between clusters were determined using a nested loop which iterates over each cluster and compares the distance with other clusters to find the minimum distance between two cluster edges. Each cluster was then assigned to its nearest neighbor with the corresponding distance, and the average mean distance was calculated from all clusters.

DNA-PAINT of insulin NanoRods

Picasso Localize was used to detect localizations by setting a minimum net gradient of 5000 and the MLE fitting algorithm with 0.001 and 1000 as criterion convergence and a maximum number of iterations. Picasso Filter was used to filter out localizations based on their localization precision error, Gaussian fitting, photons and ellipticity (Supplementary Fig. 2a). Picasso Render was used to correct sample drift by first using redundant cross-correlation (RCC) and then by using NanoRods as fiducial markers (Supplementary Fig. 2b). For RCC, a segmentation value of 1000 was used and for picking NanoRods as fiducial markers, 10 NanoRods were picked and then "picked similar" was used, followed by "undrift using picked". Docking sites were then picked as fiducial markers as previously mentioned for final drift

correction. For dual exchange-PAINT, channels were first aligned using RCC and then by picking NanoRods as fiducial markers (Supplementary Fig. 2b).

There is a possibility that DNA-PAINT underestimates ligand occupancies as the docking strand is positioned between the insulin molecule and the origami backbone (Fig. 2e) which could cause steric hindrance effects. Of note, in the gel shift assay (Fig. 2c) the bands do not broaden with increasing insulin valency, suggesting that the variability in the number of insulin molecules per NanoRod does not markedly increase with valency.

qPAINT analysis of insulin NanoRod

qPAINT was used to count the number of insulins per NanoRod (N_I) (Supplementary Fig. 3). In each condition, 100 NanoRods with a known N_I were picked by visual inspection and used to calibrate the influx rate ξ (number of blinks per frame) on Picasso Render. Then, NanoRods were picked and the N_I was calculated based on the equation $N_I = (\xi \times \tau_{d*})^{-1}$ where τ_{d*} corresponds to the mean dark time. Properties of selected NanoRods were then saved for further analysis. To quantify the N_I in NR-1 structures, dual exchange-PAINT was used (Supplementary Fig. 3b). First, NanoRods with two end spots were picked by using the image rendering of NanoRods with DS2 ends. In each picked spot, the N_I was then calculated by using the image rendering of NanoRods with insulin-DS1. To quantify the N_I in NR-15 structures, NanoRods with four DS1 were added to the samples and picked to calibrate the influx rate (Supplementary Fig. 3d).

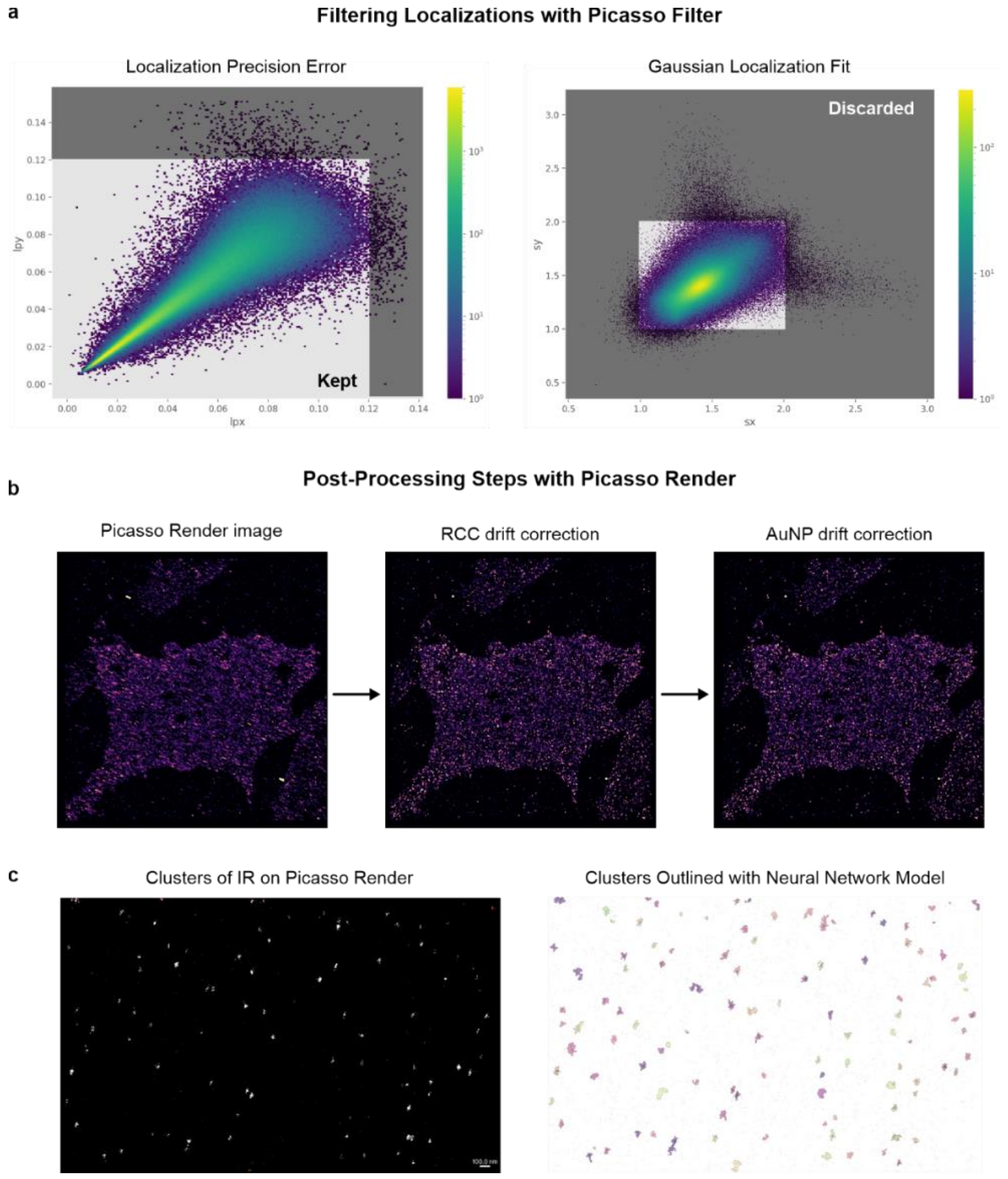

Supplementary Fig. 1. Processing and analysis steps in DNA-PAINT experiments. a, Filtering steps to remove localizations with a localization precision error higher than 10 nm and eliminate double and spurious binding events. **b,** Drift correction was done first by using redundant cross-correlation (RCC) followed by picking gold nanoparticles as fiducial markers. **c,** Images of insulin receptor clusters in Picasso Render and their outlines using the trained neural network model 87B144.

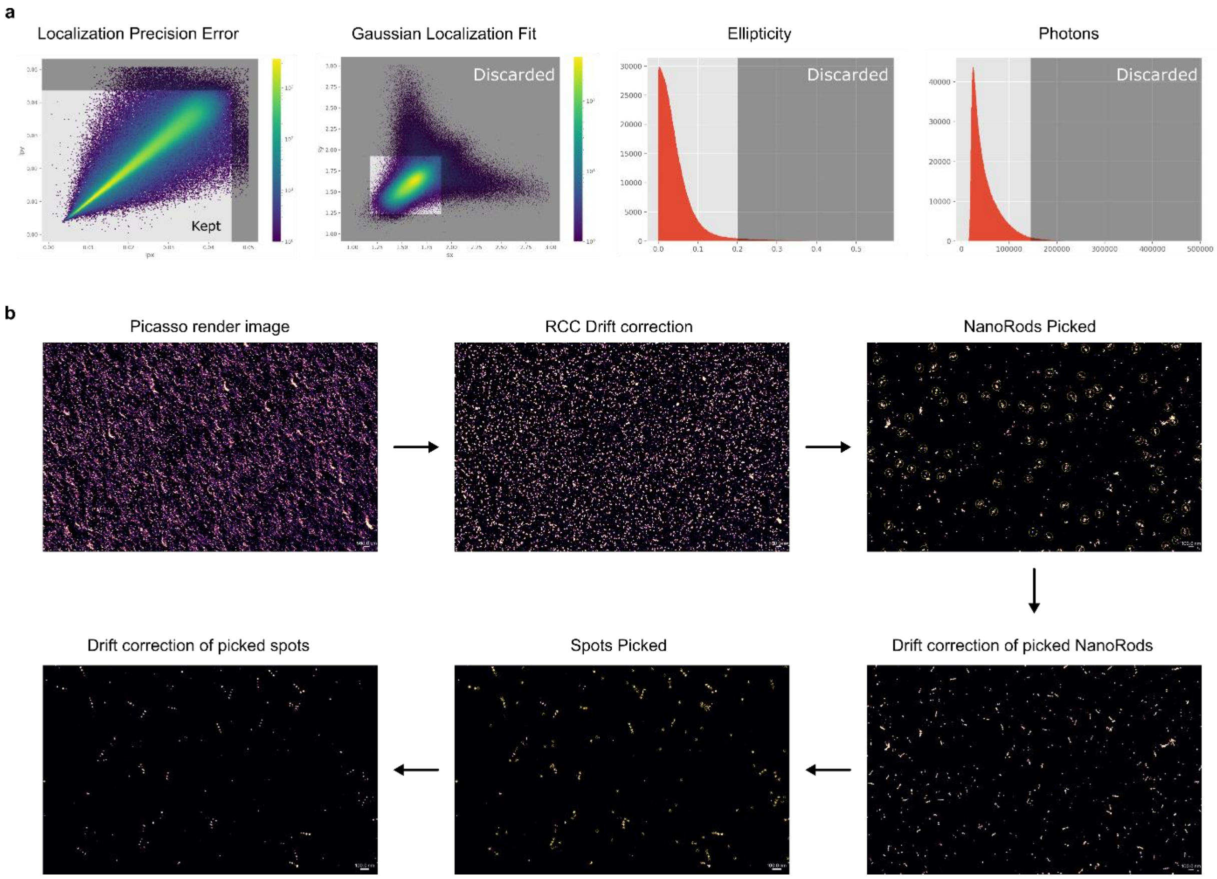

Supplementary Fig. 2. Characterization of the number of insulin molecules bound to the insulin NanoRods by DNA-PAINT. a, Filtering steps used to remove localizations with a localization precision error higher than 4 nm and eliminate double and spurious binding events. **b,** Drift correction was done in three rounds, first using redundant cross correlation (RCC) followed by two rounds using NanoRods and then single spots as fiducial markers.

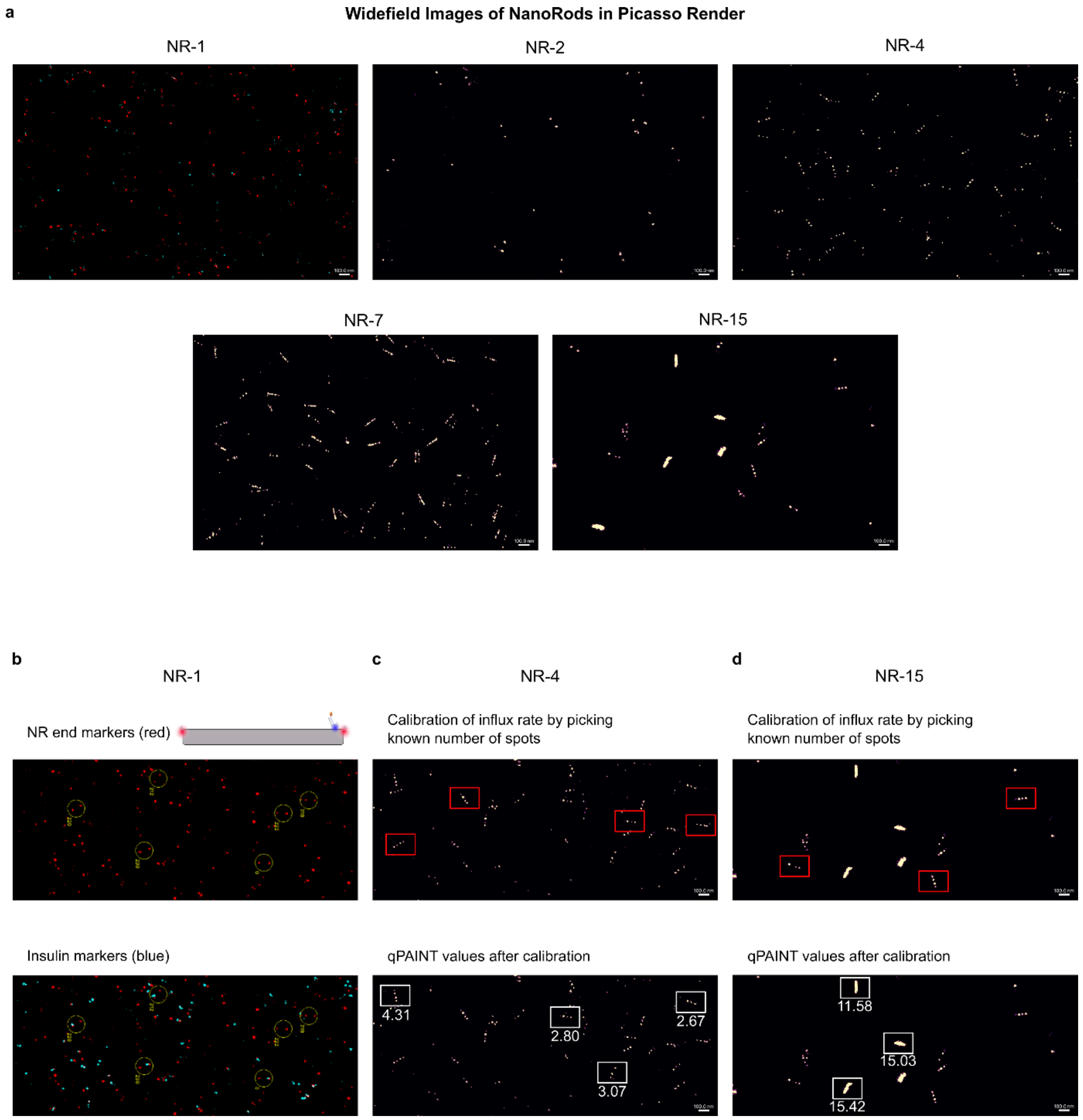

Supplementary Fig. 3. DNA-PAINT images of insulin NanoRods. a, DNA-PAINT images of NR-1, NR-2, NR-4, NR-7, and NR-15 in Picasso Render. Scale bar 100 nm. **b,** qPAINT calibration of NR-1 using end markers (see Methods for full description). **c,** qPAINT calibration of NR-4 (same protocol for NR-2 and NR-7, see Methods for full description). **d,** qPAINT calibration of NR-15 using NR-4 calibration standards (see Methods for full description). Images shown depict a representative area of one 20x20µm image from one experiment. n=450 (NR-1), n=571 (NR-2), n=584 (NR-4), n=492 (NR-7), n=535 (NR-15) structures used for quantification.

References

1. Williamson, D. J. *et al.* Machine learning for cluster analysis of localization microscopy data. *Nat. Commun.* **11**, 1493 (2020).
2. Simoncelli, S. *et al.* Multi-color Molecular Visualization of Signaling Proteins Reveals How C-Terminal Src Kinase Nanoclusters Regulate T Cell Receptor Activation. *Cell Rep.* **33**, 108523 (2020).
