# Supplementary material for: Multivalent insulin receptor activation using insulin–DNA origami nanostructures
Source: Nat Nanotechnol. 2023 Oct 9;19(2):237–45. doi: 10.1038/s41565-023-01507-y (PMC10873203; doi:10.1038/s41565-023-01507-y)
Supplement: Supplementary file 2 — Reporting Summary [file 41565_2023_1507_MOESM2_ESM.pdf]

nature portfolio

Corresponding author(s): Ana I. Teixeira

Last updated by author(s): Jul 8, 2023

Reporting Summary

Nature Portfolio wishes to improve the reproducibility of the work that we publish. This form provides structure for consistency and transparency in reporting. For further information on Nature Portfolio policies, see our Editorial Policies and the Editorial Policy Checklist.

Statistics

For all statistical analyses, confirm that the following items are present in the figure legend, table legend, main text, or Methods section.

n/a	Confirmed	
☐	☒	The exact sample size (*n*) for each experimental group/condition, given as a discrete number and unit of measurement
☐	☒	A statement on whether measurements were taken from distinct samples or whether the same sample was measured repeatedly
☐	☒	The statistical test(s) used AND whether they are one- or two-sided<br>*Only common tests should be described solely by name; describe more complex techniques in the Methods section.*
☒	☐	A description of all covariates tested
☐	☒	A description of any assumptions or corrections, such as tests of normality and adjustment for multiple comparisons
☐	☒	A full description of the statistical parameters including central tendency (e.g. means) or other basic estimates (e.g. regression coefficient) AND variation (e.g. standard deviation) or associated estimates of uncertainty (e.g. confidence intervals)
☐	☒	For null hypothesis testing, the test statistic (e.g. *F*, *t*, *r*) with confidence intervals, effect sizes, degrees of freedom and *P* value noted<br>*Give P values as exact values whenever suitable.*
☒	☐	For Bayesian analysis, information on the choice of priors and Markov chain Monte Carlo settings
☒	☐	For hierarchical and complex designs, identification of the appropriate level for tests and full reporting of outcomes
☒	☐	Estimates of effect sizes (e.g. Cohen's *d*, Pearson's *r*), indicating how they were calculated

Our web collection on statistics for biologists contains articles on many of the points above.

Software and code

Policy information about availability of computer code

Data collection

For DNA-PAINT experiments imaging was carried out out on a Nikon Eclipse Ti-E microscope with Perfect Focus system (Nikon Instruments), applying an objective-type TIRF configuration using an iLAS2 circular TIRF module (Gataca Systems). Fluorescence light was spectrally filtered with an emission filter (ET595/50m, Chroma Technology) and imaged on an iXon Ultra 888 EMCCD camera (Andor). Micromanager software v1.4 was used to acquire 12000 frames with 10 MHz readout frame.
For AFM analysis, imaging was performed using NanoWizard 3 Ultra AFM (JPK Instruments).
For TEM experiments, imaging was performed on a Talos 120C G2.
DLS experiments were performed on a Zetasizer Ultra (Malvern Panalytical).
Imaging of agarose gels was performed using an ImageQuant LAS 4000 gel imager (GE).
HPLC purification was performed using an ÄKTA Ettan LC HPLC (Amersham Pharmacia Biotech)
For SPR experiments, data was acquired using Biacore T200 System Control software v.2.01.
For western blot analysis, HRP signal detection using ChemiDoc Imaging System (Biorad).
Flow cytometry measurements performed on a BD FACSCANTO II with a BD FACSDIVA software v.9.0 (BD Biosciences)
For RNA-seq experiments, library size and quality were assessed using a Bioanalyzer 2100. Sequencing was performed using single-end reads (1x75 bp) with NextSeq 500/550 High Output Kit v2.5 on a NextSeq 550 platform (Illumina).
Confocal image acquisition performed on a Leica TCS SP8 microscope with LAS X software (v.3.5.5.19976).

Data analysis

GraphPad Prism v9.4.0, v9.3.1, oxDNA coarse-grained modelling (https://oxdna.org/), oxView tool (https://oxdna.org/), tacoxDNA (http://tacoxdna.sissa.it/), BIAevaluation v3.0, FlowJo v10.7.1 (BD Biosciences), ImageJ/Fiji v1.53, Salmon v1.7.0, DESeq2 package v.1.34.0, ComplexHeatmap v. 2.10.0, clusterProfiler v. 4.2.2, Picasso (https://github.com/jungmannlab/picasso), trained neural network model 87B144 (https://gitlab.com/quokka79/caml), custom code (https://github.com/TeixeiraLab/Spratt-et-al.)

For manuscripts utilizing custom algorithms or software that are central to the research but not yet described in published literature, software must be made available to editors and reviewers. We strongly encourage code deposition in a community repository (e.g. GitHub). See the Nature Portfolio guidelines for submitting code & software for further information.

Data

Policy information about availability of data

All manuscripts must include a data availability statement. This statement should provide the following information, where applicable:

- Accession codes, unique identifiers, or web links for publicly available datasets
- A description of any restrictions on data availability
- For clinical datasets or third party data, please ensure that the statement adheres to our policy

Source data are provided with this paper. RNA-seq data are available through the ArrayExpress database (https://www.ebi.ac.uk/biostudies/arrayexpress) under accession number E-MTAB-12160. Reference Mus musculus protein-coding transcriptome release M29 (GRCm39) (https://www.gencodegenes.org/mouse/).

Research involving human participants, their data, or biological material

Policy information about studies with human participants or human data. See also policy information about sex, gender (identity/presentation), and sexual orientation and race, ethnicity and racism.

Reporting on sex and gender	n/a
Reporting on race, ethnicity, or other socially relevant groupings	n/a
Population characteristics	n/a
Recruitment	n/a
Ethics oversight	n/a

Note that full information on the approval of the study protocol must also be provided in the manuscript.

Field-specific reporting

Please select the one below that is the best fit for your research. If you are not sure, read the appropriate sections before making your selection.

☒ Life sciences ☐ Behavioural & social sciences ☐ Ecological, evolutionary & environmental sciences

For a reference copy of the document with all sections, see nature.com/documents/nr-reporting-summary-flat.pdf

Life sciences study design

All studies must disclose on these points even when the disclosure is negative.

Sample size	No statistical methods were used to pre-determine sample sizes, but our sample sizes were similar to those reported in previous publications (PMID:22608007, 24904114, 5177603). All sample sizes are listed in each figure legend.
Data exclusions	No data were excluded from analysis
Replication	Reproducibility was ensured by sampling from multiple biological replicates, i.e., from multiple adipocyte differentiation cultures or from different ablation experiments in zebrafish larva. The exact number of independent biological experiments or number of lavae used for each measurement is mentioned in the figure legends. No results are included that were not observed in multiple experiments. All attempts at replication were successful.
Randomization	For experiments with adipocyte cultures, cells were visually analysed to assess cell surface density and to evaluate differentiation of cells, and then randomly allocated to treatment and control groups. For SPR experiments randomization was not applicable, since they cannot be allocated into different experimental groups. Zebrafish larvae were randomly assigned to treatment groups.
Blinding	Investigators were not blind to subject groups because knowledge of experimental conditions was required during data collection and evaluation.

Reporting for specific materials, systems and methods

We require information from authors about some types of materials, experimental systems and methods used in many studies. Here, indicate whether each material, system or method listed is relevant to your study. If you are not sure if a list item applies to your research, read the appropriate section before selecting a response.

Materials & experimental systems

n/a	Involved in the study	
☐	☒	Antibodies
☐	☒	Eukaryotic cell lines
☒	☐	Palaeontology and archaeology
☐	☒	Animals and other organisms
☒	☐	Clinical data
☒	☐	Dual use research of concern
☒	☐	Plants

Methods

n/a	Involved in the study	
☒	☐	ChIP-seq
☐	☒	Flow cytometry
☒	☐	MRI-based neuroimaging

Antibodies

Antibodies used

Antibodies/Supplier/Catalog No / Dilution
rabbit monoclonal anti-phosho-IR-IGF1R (19H7) / Cell Signaling Technology/ 3024 / 1:1000 (WB)
rabbit monoclonal anti-phospho-AKT (193H12)/ Cell Signaling Technology/ 4058/ 1:1000 (WB)
rabbit monoclonal anti-Insulin Receptor β (4B8)/ Cell Signaling Technology/ 3025 /1:300 (DNA-PAINT)
rabbit policlonal anti-GAPDH/ Invitrogen/ PA1-987/ 1:5000 (WB)
goat anti-rabbit IgG, HRP conjugated/ Invitrogen/ 31460/ 1:5000
camelid anti-rabbit IgG/ Massive Photonics/ 1:200

Validation

Previously published antibodies or antibodies with company based validations were used:

rabbit anti-phosho-IR-IGF1R (CST,3024): https://www.cellsignal.com/products/primary-antibodies/phospho-igf-i-receptor-b-tyr1135-1136-insulin-receptor-b-tyr1150-1151-19h7-rabbit-mab/3024

rabbit anti-phospho-AKT (CST,4058): https://www.cellsignal.com/products/primary-antibodies/phospho-akt-ser473-193h12-rabbit-mab/4058

rabbit anti-Insulin Receptor β (CST, 3025): https://www.cellsignal.com/products/primary-antibodies/insulin-receptor-b-4b8-rabbit-mab/3025; Dall'Agnese et al. Nat. Commun, 2022 (PMID: 36473871)

rabbit anti-GAPDH (Invitrogen,PA1-987): https://www.thermofisher.com/antibody/product/GAPDH-Antibody-Polyclonal/PA1-987

Additionally, for western blots experiments, samples of cells treated with or without insulin after serum starvation treatment were used to validate phospho-IR and phospho-AKT antibodies.

Eukaryotic cell lines

Policy information about cell lines and Sex and Gender in Research

Cell line source(s)

An immortalized brown preadipocyte mouse cell line generated in Dr. Bruce Spiegelman´s lab was used for differentiation of adipocytes. This cell lines has been previously described in: Uldry, M. et al. Cell Metab., 2006 (PMID: 16679291)

Authentication

Cell lines were authenticated by providers. We further authenticated this cell line by testing its ability to differentiate to adipocytes by evaluating their morphology, accumulation of lipids, and by qPCR analysis of fat-selective genes using species specific primers.

Mycoplasma contamination

Cell line was tested negative for mycoplasma contamination using LookOut Mycoplasma qPCR detection kit (Sigma)

Commonly misidentified lines
(See ICLAC register)

No commonly misidentified cell lines were used

Animals and other research organisms

Policy information about studies involving animals; ARRIVE guidelines recommended for reporting animal research, and Sex and Gender in Research

Laboratory animals

The following zebrafish lines were used: Tg(ins:CFP-NTR) and Tg(ins:CFP-NTR);Tg(ins:Kaede). All zebrafish experiments were performed on larvae (up to 3dpf). Larvae were two days old at start of experiments .

Wild animals	This study did not involve wild animals
Reporting on sex	Sex was not considered in the design of experiments since experiments were carried out at early larvae stages before initiation of sexual differentiation. Larvae were selected randomly to different treatment groups.
Field-collected samples	This study did not involve field-collected samples
Ethics oversight	Maintenance and crossing of zebrafish lines were conducted in compliance with Swedish legislation on animal welfare regulations approved by Stockholms djurförsöksetiska nämnd. Since for β-cell ablation and free glucose assay experiments only animals younger than 5 days were used, no ethical permit was required according to 2010/63/EU.

Note that full information on the approval of the study protocol must also be provided in the manuscript.

Flow Cytometry

Plots

Confirm that:

☒ The axis labels state the marker and fluorochrome used (e.g. CD4-FITC).

☒ The axis scales are clearly visible. Include numbers along axes only for bottom left plot of group (a 'group' is an analysis of identical markers).

☒ All plots are contour plots with outliers or pseudocolor plots.

☒ A numerical value for number of cells or percentage (with statistics) is provided.

Methodology

Sample preparation	Differentiated adipocytes were dissociated in collagenase D solution, resuspended in staining buffer (1x PBS, 1% BSA). Dead cells were labelled with LIVE/DEAD™ Fixable Yellow Dead Cell Stain Kit and incubated with 10 nM ATTO-647 labelled NanoRod structures for 10 mins at 37°C. Cells incubated without NanoRod structures were used as untreated control. Cells were then washed twice with staining buffer.
Instrument	BD FACSCANTO II
Software	BD FACSDIVA software (BD Biosciences); FlowJo 10.7.1 Software (BD Biosciences)
Cell population abundance	Experiments were aimed at determing binding of insulin NanoRod structures to adipocyte cells from in vitro differentiation of pre-adipocyte cell line. Based on analysis of accumulation of lipid droplets in cells the percentage of cells differentiating to adipocyte cells is high.
Gating strategy	Live adipocyte cells were initially identified by gating cells on FSC-A vs AmCyan-A, followed by gating the FSC-A vs FSC-H to detect singlets.

☒ Tick this box to confirm that a figure exemplifying the gating strategy is provided in the Supplementary Information.
