# Supplementary figures and images for: Multivalent insulin receptor activation using insulin–DNA origami nanostructures
Source: Nat Nanotechnol. 2023 Oct 9;19(2):237–45. doi: 10.1038/s41565-023-01507-y (PMC10873203; doi:10.1038/s41565-023-01507-y)

## Source Data for Fig.2

Fig. 2c

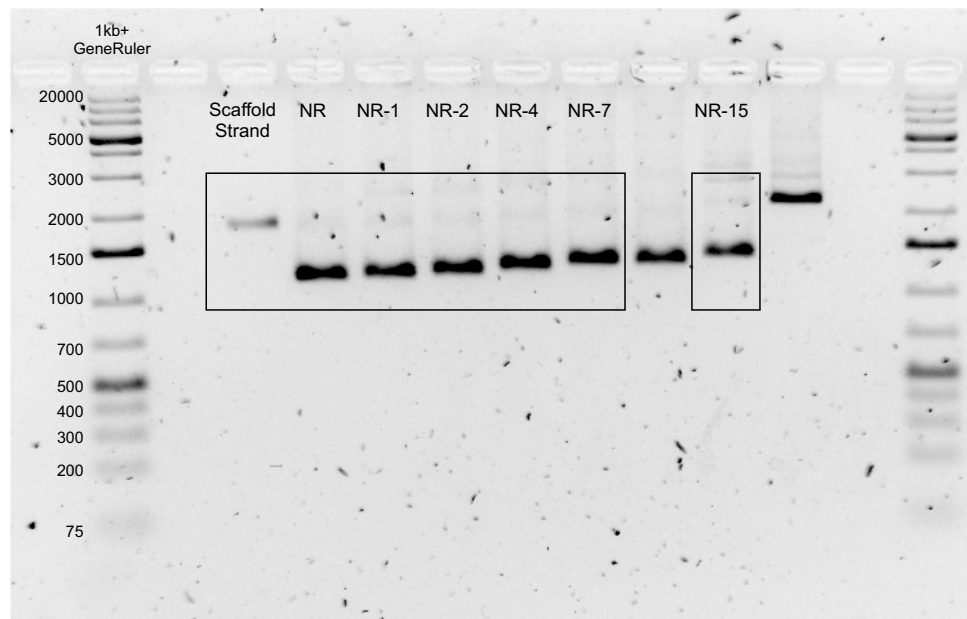

Supplement: Supplementary file 6 — Unprocessed gels. [file 41565_2023_1507_MOESM6_ESM.pdf]

## Source Data for Extended Data Fig. 2

Extended Data Fig. 2d

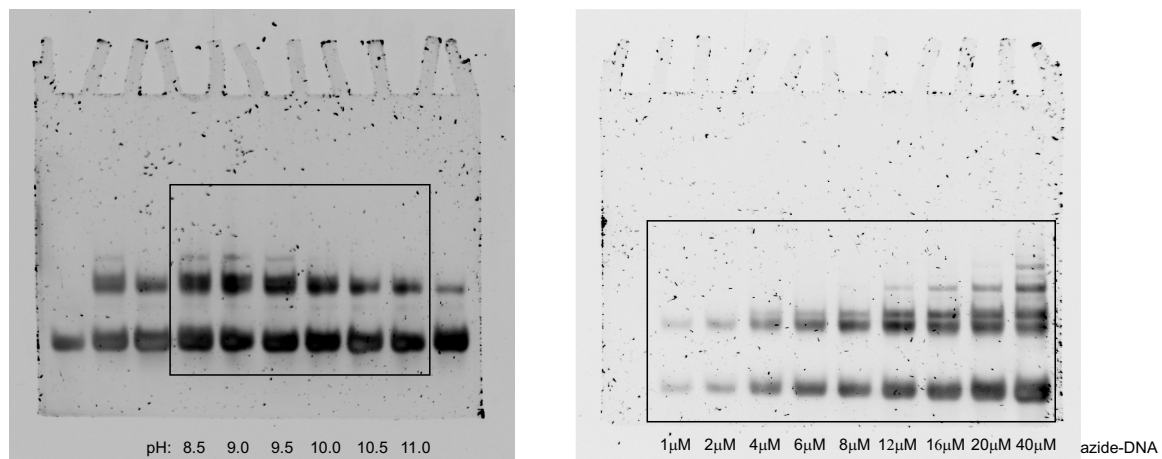

Extended Data Fig. 2e

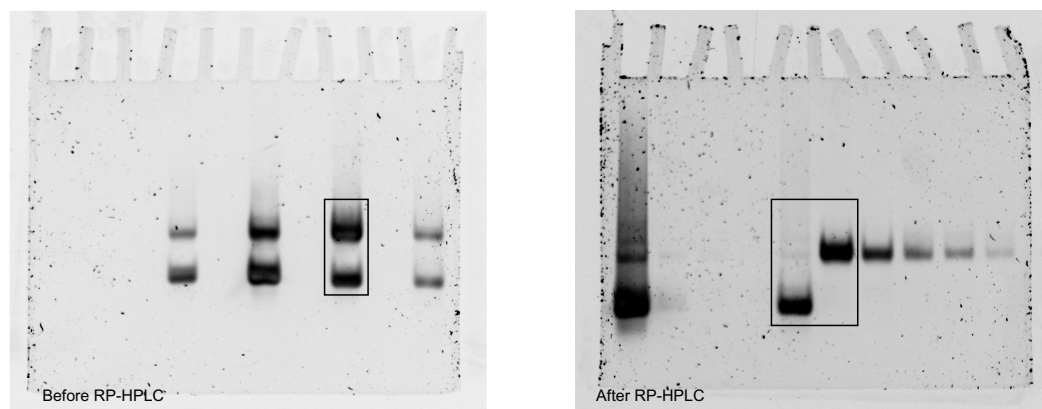

Supplement: Supplementary file 13 — Unprocessed gels. [file 41565_2023_1507_MOESM13_ESM.pdf]

## Source Data for Extended Data Fig. 4

Extended Data Fig. 4f

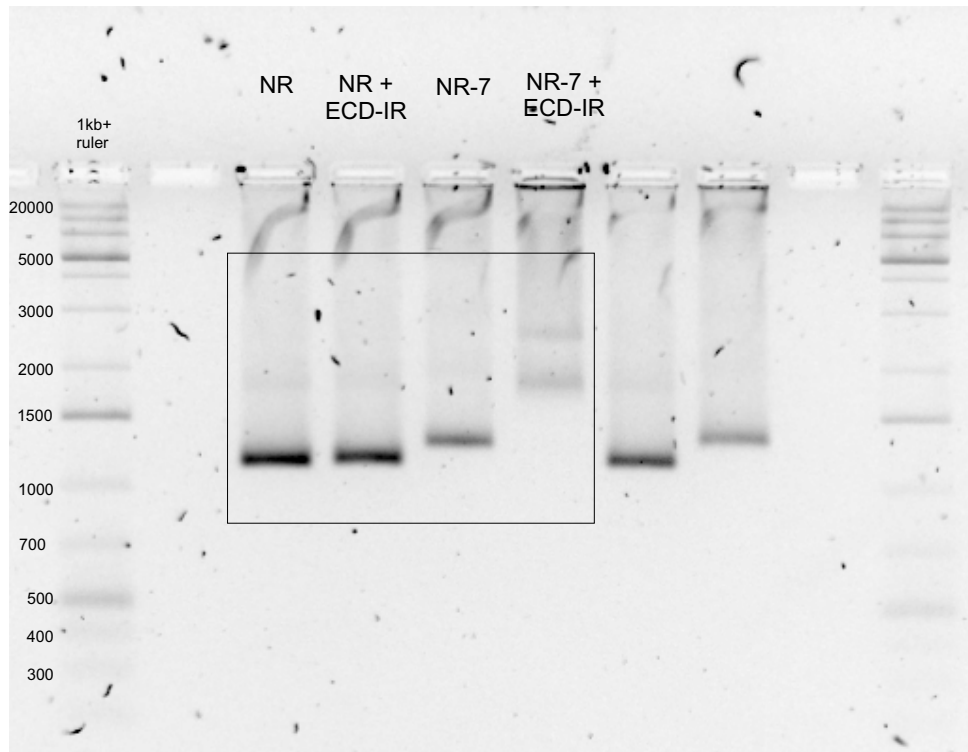

Supplement: Supplementary file 15 — Unprocessed gels. [file 41565_2023_1507_MOESM15_ESM.pdf]
