# Supplementary material for: Multivalent insulin receptor activation using insulin–DNA origami nanostructures
Source: Nat Nanotechnol. 2023 Oct 9;19(2):237–45. doi: 10.1038/s41565-023-01507-y (PMC10873203; doi:10.1038/s41565-023-01507-y)
Supplement: Supplementary file 9 — Unprocessed western blots. [file 41565_2023_1507_MOESM9_ESM.pdf]

Source Data for Fig.4

Fig. 4b

Colorimetric image of membrane | imaging of all samples | imaging without last sample

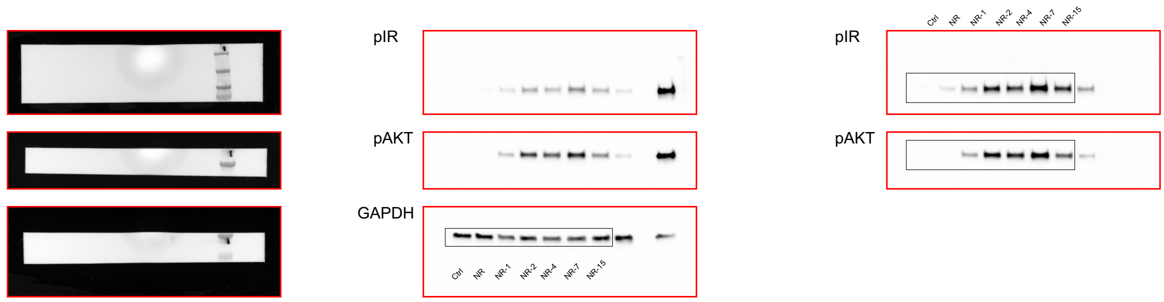

Membranes were cut before incubation with primary antibody using the pre-stained molecular weights as a guide.
Membranes were also imaged (”Colorimetric image of membrane”).
Blots used for detection of pIR and pAKT signal contained additional samples not included in the study.
Detection was done with all samples (”imaging of all samples”) or where the last sample was covered before imaging (”Imaging without last sample).
Bands presented in Fig. 4b are indicated by black boxes.

Fig. 4e

Colorimetric image of membrane

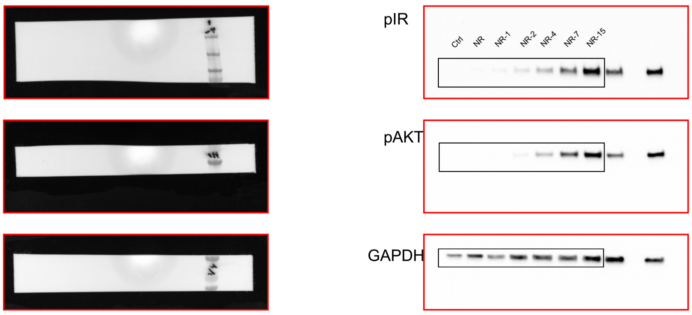

Membranes were cut before incubation with primary antibody using the pre-stained molecular weights as a guide.
Membranes were also imaged (”Colorimetric image of membrane”).
Bands presented in Fig. 4e are indicated by black boxes.

Fig. 4i

Colorimetric image of membrane | imaging of all samples | imaging without extra sample

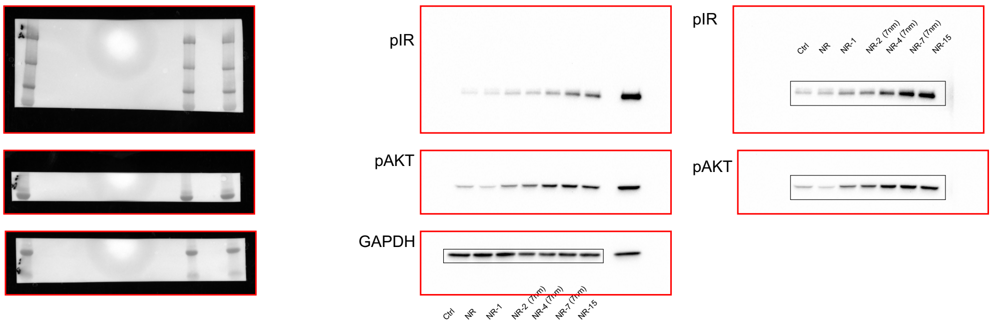

Membranes were cut before incubation with primary antibody using the pre-stained molecular weights as a guide.
Membranes were also imaged (”Colorimetric image of membrane”).
Blots used for detection of pIR and pAKT signal contained additional samples not included in the study.
Detection was done with all samples (”imaging of all samples”) or where the extra samples were covered before imaging (”Imaging without extra sample).
Bands presented in Fig. 4i are indicated by black boxes.
