# Supplementary material for: Multivalent insulin receptor activation using insulin–DNA origami nanostructures
Source: Nat Nanotechnol. 2023 Oct 9;19(2):237–45. doi: 10.1038/s41565-023-01507-y (PMC10873203; doi:10.1038/s41565-023-01507-y)
Supplement: Supplementary file 21 — Unprocessed western blots. [file 41565_2023_1507_MOESM21_ESM.pdf]

Source Data for Extended Data Fig. 9

Extended Data Fig. 9d

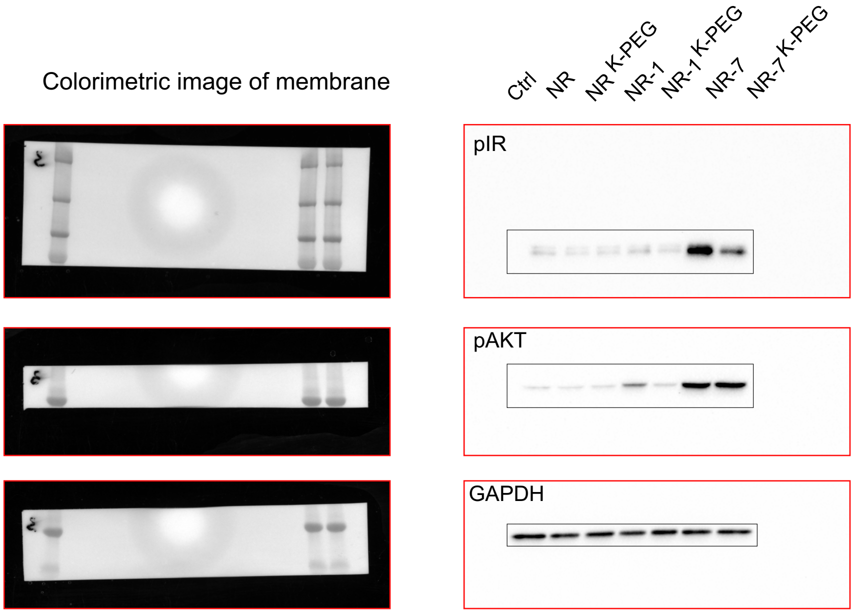

Membranes were cut before incubation with primary antibody using the pre-stained molecular weights as a guide.
Membranes were also imaged ("Colorimetric image of membrane").
Bands presented in Extended Data Fig. 5a are indicated by black boxes.
